# SHP2 is a multifunctional therapeutic target in drug resistant metastatic breast cancer

**DOI:** 10.1038/s41388-020-01488-5

**Published:** 2020-10-08

**Authors:** Hao Chen, Sarah Libring, Kasi Viswanatharaju Ruddraraju, Jinmin Miao, Luis Solorio, Zhong-Yin Zhang, Michael K. Wendt

**Affiliations:** 1 Department of Medicinal Chemistry and Molecular Pharmacology, Purdue University, West Lafayette, IN 47907 USA; 2 Department of Biomedical Engineering, Purdue University, West Lafayette, IN 47907 USA; 3 Purdue University Center for Cancer Research, Purdue University, West Lafayette, IN 47907 USA

**Keywords:** Breast cancer, Mechanisms of disease, Target validation

## Abstract

Metastatic breast cancer (MBC) is an extremely recalcitrant disease capable of bypassing current targeted therapies via engagement of several growth promoting pathways. SH2 containing protein tyrosine phosphatase-2 (SHP2) is an oncogenic phosphatase known to facilitate growth and survival signaling downstream of numerous receptor inputs. Herein, we used inducible genetic depletion and two distinct pharmacological inhibitors to investigate the therapeutic potential of targeting SHP2 in MBC. Cells that acquired resistance to the ErbB kinase inhibitor, neratinib, displayed increased phosphorylation of SHP2 at the Y542 activation site. In addition, higher levels of SHP2 phosphorylation, but not expression, were associated with decreased survival of breast cancer patients. Pharmacological inhibition of SHP2 activity blocked ERK1/2 and AKT signaling generated from exogenous stimulation with FGF2, PDGF, and hGF and readily prevented MBC cell growth induced by these factors. SHP2 was also phosphorylated upon engagement of the extracellular matrix (ECM) via focal adhesion kinase. Consistent with the potential of SHP2-targeted compounds as therapeutic agents, the growth inhibitory property of SHP2 blockade was enhanced in ECM-rich 3D culture environments. In vivo blockade of SHP2 in the adjuvant setting decreased pulmonary metastasis and extended the survival of systemic tumor-bearing mice. Finally, inhibition of SHP2 in combination with FGFR-targeted kinase inhibitors synergistically blocked the growth of MBC cells. Overall, our findings support the conclusion that SHP2 constitutes a shared signaling node allowing MBC cells to simultaneously engage a diversity of growth and survival pathways, including those derived from the ECM.

## Introduction

Stage IV, metastatic breast cancer (MBC) is a major clinical challenge, with 5 year survival rates currently at ~23% [1]. Tyrosine kinase inhibitors targeting human epidermal growth factor receptor 2 (HER2), such as lapatinib and neratinib, can provide therapeutic benefit to HER2^+^ patients. However, inherent and acquired drug resistance inevitably occur in the metastatic setting, leading to further disease progression [2–6]. Given the relationship between drug resistance and metastasis, there exists the possibility that the underlying drivers of these processes may be shared. Identification and pharmacological inhibition of the shared targets of drug resistance and metastasis remains a major barrier to the development of effective therapeutics for the treatment of late-stage disease.

Multiple experimental studies, including findings from our lab, describe a role for fibroblast growth factor receptor 1 (FGFR1) in acquired and inherent resistance to HER2-targeted therapeutics [7–10]. The locus encoding *FGFR1* is amplified in 14% of breast cancer patients, and FGFR1 expression can be further upregulated through the process of epithelial–mesenchymal transition, a key driver of both drug resistance and metastasis [11, 12]. In addition to FGFR, multiple other receptor tyrosine kinases (RTKs) have been linked to both drug resistance and metastasis, including PDGFR, VEGFR, EGFR, and cMET [13–15]. Finally, active signaling events generated through integrin-mediated sensing of the extracellular matrix (ECM) can function independently and in conjunction with RTKs to support the growth and survival of MBC cells in the presence of targeted therapies and chemotherapies [16, 17]. Therefore, we sought to identify signaling nodes shared between multiple RTKs and ECM signaling events as targeting these nodes may hold the key to the development of MBC therapies.

Src homology region 2 (SH2)-containing protein tyrosine phosphatase-2 (SHP2) is an oncogenic tyrosine phosphatase involved in downstream signaling of several RTKs, and was therefore chosen for analysis as a shared signaling node [18–21]. SHP2 exists in an inactivated form due to an intramolecular allosteric interaction between the N-terminal SH2 domain and the C-terminal phosphatase domain. Phosphorylation at Y542 releases this inhibition and the molecule unfolds, becoming enzymatically active [22, 23]. The phosphatase activity of SHP2 is clearly required for critical cancer-associated signaling pathways such as RAS and PI3K, but the biochemical mechanisms of how SHP2 supports oncogenic signaling still remain to be definitively determined [24, 25].

Two types of small molecule inhibitors of SHP2 have recently emerged. SHP099 is an allosteric inhibitor of SHP2, and we have recently developed 11a-1, as an active-site inhibitor of SHP2 [26–28]. Genetic deletion and pharmacological inhibition of SHP2 have been reported to block the growth of primary tumors in breast cancer xenograft and genetically engineered mouse models [29–32]. Given recent findings around the function of SHP2, and the established pharmacological tools to therapeutically inhibit its activity, we sought to address the hypothesis that SHP2 will be an effective therapeutic target for drug-resistant MBC [33].

Herein, we demonstrate that SHP2 becomes phosphorylated through ECM signaling, correlating with enhanced efficacy of SHP2 inhibitors in 3D growth environments as compared to traditional 2D culture. In vivo, SHP2 inhibition blocked proliferation of MBC cells and delayed pulmonary metastatic progression, a result that could be enhanced by combination with FGFR-targeted kinase inhibitors. Overall, we posit that combined inhibition of FGFR and SHP2 may be an effective treatment strategy for MBC.

## Results

### SHP2 facilitates in vitro growth of metastatic breast cancer cells

**Fig. 1:**
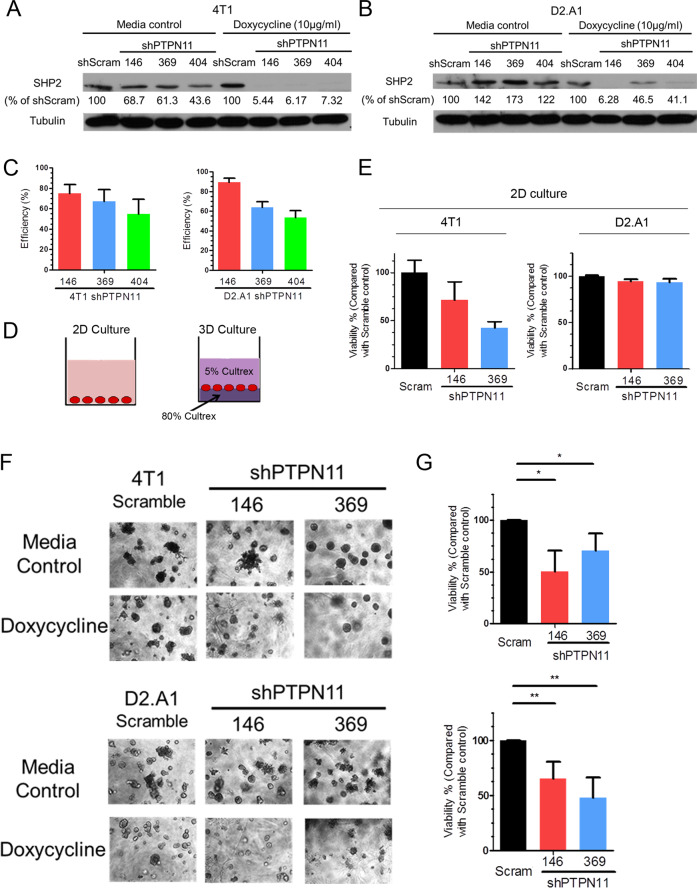
Depletion of SHP2 inhibits the growth of metastatic breast cancer cells. **a**, **b** Immunoblot analyses with quantification for SHP2 in 4T1 and D2.A1 cells stably expressing three independent doxycycline-inducible shRNA sequences targeting PTPN11 with and without doxycycline induction, compared with a scrambled (scram) shRNA control. **c** Quantification of SHP2 depletion for each shRNA construct in 4T1 and D2.A1 cells (*n* = 3). Depletion efficiency was defined as the percentage of SHP2 protein decrease normalized to scramble controls upon doxycycline induction. **d** Schematic representation of 2D and 3D growth conditions. **e** 2D growth assays showing differential cell viability of 4T1 and D2.A1 cells upon doxycycline-induced depletion of SHP2. Data are the mean ± s.e.m. of two biological replicates completed in triplicate. **f** Representative photos showing 3D morphologies of 4T1 and D2.A1 cells upon depletion of SHP2. **g** Quantification of differential cell viability of 4T1 and D2.A1 cells upon depletion of SHP2. Data are shown as mean ± s.e.m. (*n* = 3 for 4T1; *p < 0.05; *n* = 4 for D2.A1, **p < 0.01).

We used three independent doxycycline-inducible shRNAs targeting PTPN11 to manipulate SHP2 expression in two MBC cell lines. The 4T1 and D2.A1 cells are murine models of stage IV breast cancer, capable of producing systemic metastases following orthotopic engraftment of mammary fat pad tumors. Similar to human disease, previous studies from our lab and others indicate that the metastatic phenotypes in these cells are driven by a variety of RTK and ECM signaling events [12, 34–36]. Upon stable transduction with inducible shRNA expression constructs, these cells were transiently treated with doxycycline and GFP^+^ cells were isolated by FACS (Supplementary Fig. S1a–f). Using this approach we were able to deplete SHP2 levels by 79.8% in the 4T1 cells and 89.5% in the D2.A1 cells upon addition of doxycycline (Fig. 1a–c and Supplementary Fig. S1g, h). We next utilized these depletion systems to perform growth assays under 2D and 3D culture conditions. As shown in Fig. 1d, cells were either cultured directly on tissue culture polystyrene for 2D culture conditions, or cells were placed at the interface of two semi-solid layers of ECM matrix for 3D culture conditions (Fig. 1d). Depletion of SHP2 significantly inhibited the growth of the 4T1 cells under both 2D and 3D culture conditions, but the growth inhibitory effects of SHP2 depletion were only observed when D2.A1 cells were cultured under 3D conditions (Fig. 1e–g). The specific growth inhibitory effects of SHP2 depletion under 3D culture conditions led us to further investigate the functions of SHP2 in ECM signaling.

### Pharmacologic targeting of SHP2 decreases pulmonary metastasis

**Fig. 2:**
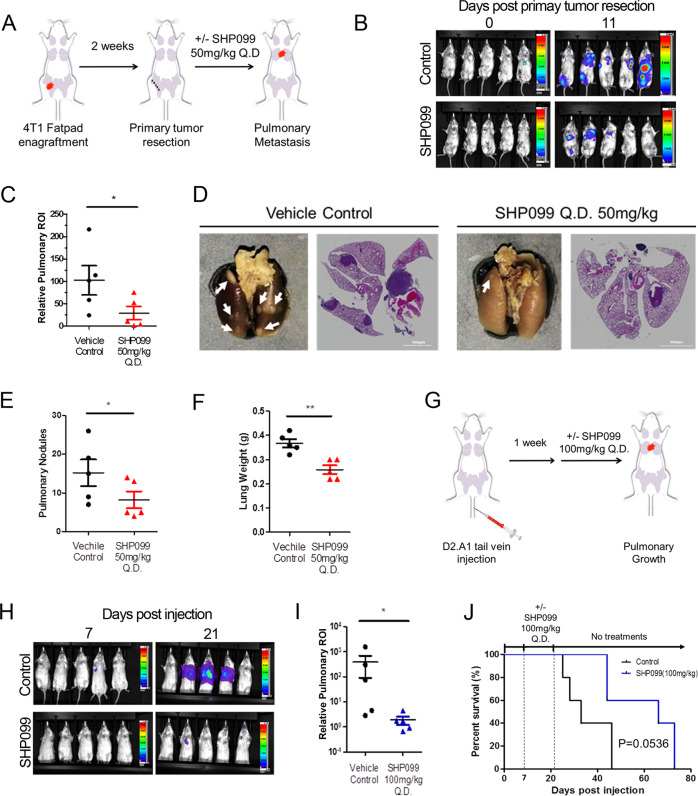
Pharmacological inhibition of SHP2 blocks in vivo pulmonary metastasis. **a** Schematic representation of the 4T1 post-surgical model of metastasis. **b** Representative bioluminescent images taken at Day 0 and Day 11 of SHP099 treatment. **c** Bioluminescent values of pulmonary regions of interest (ROI) were quantified as a measure of metastasis. Data are the ratio of luminescence values at Day 11 of treatment compared to Day 0. (*p < 0.05 for *n* = 5 mice per group). **d** Representative photos of pulmonary metastases (arrow heads) and H&E staining of pulmonary histological sections in control and SHP099 treated mice. **e, f** Plots comparing numbers of pulmonary nodules and lung weights from control and SHP099 treated mice (*p < 0.05, **p < 0.01, *n* = 5 mice per group). **g** Schematic of the D2.A1 model of pulmonary tumor growth. **h** Representative images of pulmonary growth monitored by bioluminescence at Day 7 and Day 21 post injection. **i** Bioluminescent values from pulmonary ROI quantified as the ratio of day 21 to day 7 following tumor cell injection (*p < 0.05, *n* = 5 mice per group). **j** Kaplan–Meier analyses of control and SHP099 treated mice, bearing D2.A1 pulmonary tumors, resulting in the indicated *p* value (*n* = 5 mice per group).

To further identify whether targeting SHP2 can inhibit metastatic tumor growth we implemented an adjuvant treatment protocol in conjunction with the 4T1 orthotopic model of metastasis (Fig. 2a). This approach recapitulates a surgical intervention to remove the primary tumor followed by pharmacological inhibition of residual systemic disease [37]. Following surgical resection of the primary tumor, systemic tumor bearing animals were treated with the allosteric SHP2 inhibitor, SHP099, or vehicle via oral gavage for a period of 11 days. This treatment led to a significant reduction in outgrowth of pulmonary metastases as determined by bioluminescent imaging, ex vivo quantification of pulmonary metastatic nodules, H&E staining of histological sections, and overall pulmonary weight (Fig. 2b–f).

To further determine the efficacy of SHP099 in the pulmonary microenvironment, we inoculated mice with D2.A1 cells via a tail vein injection (Fig. 2g). One week after tail vein engraftment, mice were again treated with SHP099 and pulmonary tumor growth was quantified via bioluminescence. Consistent with what we observed in the 4T1 model, 14 days of SHP099 therapy resulted in a significant decrease in pulmonary tumor burden (Fig. 2h, i). Finally, this 14-day SHP099 treatment period also extended the subsequent survival of mice as compared with the vehicle control group (Fig. 2j).

### Three dimensional culture enhances growth inhibition by SHP2 blockade

**Fig. 3:**
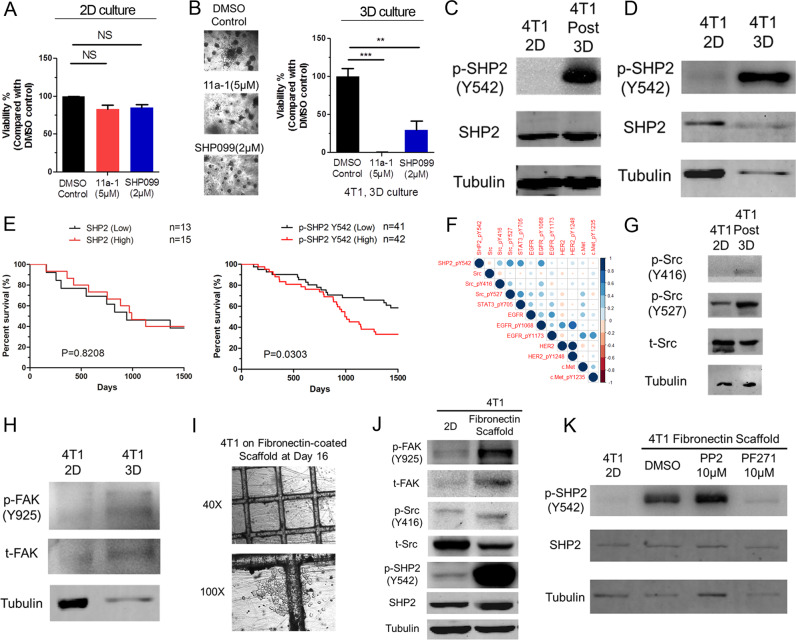
The extracellular matrix promotes SHP2 phosphorylation. **a** Cell viability of 4T1 cells cultured in 2D for 6 days in the absence or presence of the indicated concentrations of 11a-1 and SHP099. **b** Representative photomicrographs and quantification of 4T1 cell viability after 16 days in 3D culture in the absence or presence of the indicated concentrations of 11a-1 and SHP099. For **a** and **b** data are normalized to untreated controls and are the mean ± s.e.m. of three experiments where **p < 0.01 and ***p < 0.001. Immunoblot analyses showing differential phosphorylation of SHP2 at Y542 in 4T1 cells following 3D culture (**c**) or in 4T1 cells lysed directly from gel-based 3D culture (**d**). **e** Kaplan–Meier analyses of patients from the TCGA breast cancer dataset separated into two groups based on the median mRNA expression value of SHP2 (left) or median phosphorylation levels of SHP2 at Y542 (right). Overall survival was analyzed by a log-rank test resulting in the indicated *p* values. **f** RPPA data from the TCGA breast cancer dataset were analyzed for correlation of total expression levels and post translational modifications of the indicated proteins in relation to SHP2-Y542 phosphorylation. The heat map indicates the Pearson correlation coefficient and the size of the circle is representative of the value. **g** Immunoblot analyses showing differential phosphorylation of Src in 4T1 cells following culture under gel-based 3D conditions as compared to 2D culture. **h** Immunoblot analyses showing differential phosphorylation of FAK at Y925 in 4T1 cells isolated directly from gel-based 3D cultures as compared to 2D culture. **i** Representative photomicrographs showing 4T1 cells cultured on FN-coated tessellated scaffolds. **j** Immunoblot analyses showing differential phosphorylation of FAK, Src, and SHP2 in 4T1 cells cultured on FN-coated scaffolds as compared to 2D culture. **k** 4T1 cells were cultured on FN-coated scaffolds for 16 days and treated with the indicated concentrations of a Src inhibitor (PP2) or a FAK inhibitor (PF271) for the last 24 h before harvesting cells for immunoblot analyses of SHP2 phosphorylation at Y542. All immunoblots shown are representative of at least three independent experiments.

Genetic depletion of SHP2 inhibited cell growth to a greater extent under 3D culture conditions as compared to 2D culture (Fig. 1e–g). Therefore, we sought to define the impact of extracellular matrix signaling on SHP2. To pharmacologically recapitulate our results from Fig. 1, we treated 4T1 cells with two small molecule inhibitors of SHP2 under the 2D and 3D culture conditions described in Fig. 1d. Treatment with effective concentrations of either 11a-1 or SHP099 under serum containing conditions failed to significantly inhibit the growth of 4T1 cells in 2D culture (Fig. 3a). In contrast, use of these SHP2 inhibitors at the same concentrations significantly decreased the growth of 4T1 cells in 3D culture (Fig. 3b). Similar results were also observed in several other MBC cell lines (Supplementary Fig. S2).

Given this enhanced reliance of cells on SHP2 for growth in 3D culture we next assessed changes in SHP2 phosphorylation upon cellular engagement of the ECM [22]. Phosphorylation of SHP2 at Y542 was clearly enhanced when 4T1 cells were cultured under 3D conditions (Fig. 3c, d). We also observed induction of SHP2 Y542 phosphorylation in D2.A1 cells upon 3D culture and in 4T1 ex vivo lysates derived from lung metastases (Supplementary Fig. S3a, b). Consistent with these data, analysis of the TCGA dataset indicated differential expression levels of SHP2 were not predictive of patient survival, but elevated phosphorylation of SHP2 at Y542 was associated with decreased breast cancer patient survival (Fig. 3e). Further analysis of the TCGA dataset demonstrated a positive correlation between SHP2 Y542 phosphorylation and that of EGFR, STAT3, and Src phosphorylation (Fig. 3f). As Src is a key enzyme in ECM signaling, we focused on characterizing the relationship between Src and SHP2. Similar to SHP2, phosphorylation of Src at Y416 was increased in 4T1 cells following 3D culture (Fig. 3g). We also noted that 3D culture conditions enhanced phosphorylation of focal adhesion kinase (FAK) (Fig. 3h). Therefore, we next investigated whether Src and FAK contributed to SHP2 phosphorylation. To do this we utilized our recently developed tessellated scaffold system [38]. In the gel-based 3D culture system described previously ECM proteins are largely in a globular form. However, our tessellated system allows for creation of fibular networks of ECM matrixes that are more characteristic of the in vivo condition. In addition, the absence of gel allows for more efficient cell recovery, making the system more suitable for signaling analyses (Fig. 3i). Using this culture system, we found that phosphorylation of SHP2 at Y542 was elevated upon engagement of fibrillar fibronectin. This event could be ablated by addition of the FAK inhibitors PF-562271 (PF271) or defactinib, but not by the Src inhibitor PP2 (Fig. 3j, k and Supplementary Fig. S3e). We similarly observed FAK-dependent phosphorylation of SHP2 in the D2.A1 cell line when cultured on fibrillar fibronectin (Supplementary Fig. S3c, d, f, g). In summary, these data suggest that phosphorylation of SHP2 at Y542 is associated with disease progression and that this event can be mediated by the ECM in a FAK-dependent manner.

### Inhibition of SHP2 targets the growth of drug resistant HER2^+^ breast cancer cells

**Fig. 4:**
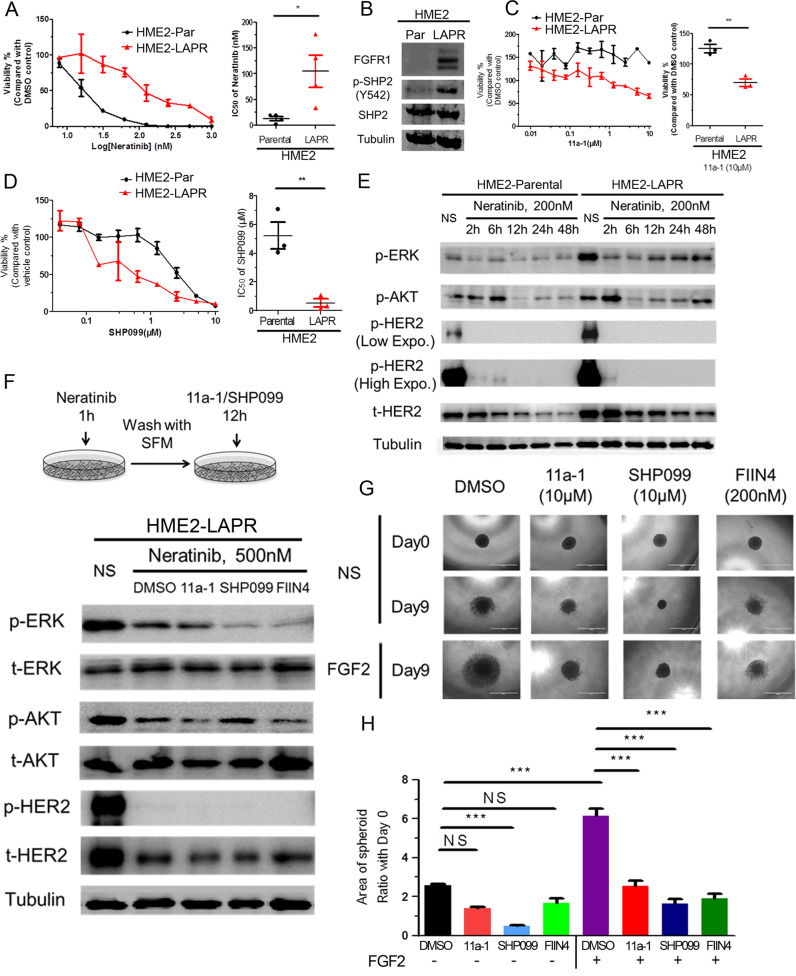
Drug resistant breast cancer cells can be targeted by inhibition of SHP2. **a** Representative dose response of HME2 parental (HME2-Par) and lapatinib resistant (HME2-LAPR) cells treated with neratinib for 6 days (right). The IC_50_ values for each independent experiment were calculated (*n* = 4) and analyzed using a two-tail student *t* test where *p < 0.05. **b** Immunoblot analyzes showing differential expression of FGFR1 and phosphorylation of SHP2 at Y542 in HME2-LAPR cells compared to their HME2-Par counterparts. **c** Representative dose response upon 6 days treatment with 11a-1. (right) Cell viability upon treatment with 10 μM 11a-1 for each independent experiment was calculated (*n* = 4) and analyzed using a two-tail student *t* test where **p < 0.01. **d** Representative dose response curves of HME2 parental (HME2-Par) and HME2-LAPR cells treated with SHP099 for 6 days. (right) The IC_50_ values for each independent experiment were calculated (*n* = 3) and analyzed using a two-tail student *t* test where **p < 0.01. **e** Immunoblotting showing phosphorylation of AKT, ERK1/2, and HER2 in HME2-Par and HME2-LAPR cells upon the indicated neratinib treatments. **f** (top) Schematic representation of the signaling recovery assays. HME2-LAPR cells were treated with neratinib for 1 h, the drug was removed, and the cells were allowed to recover in serum free media in the presence or absence of SHP2 or FGFR inhibitors for 12 h. DMSO was used as a vehicle control. (bottom) Recovery of AKT, ERK1/2, and HER2 phosphorylation were analyzed by immunoblot. **g, h** HME2-LAPR spheres were formed in a round bottom plate and then plated onto a bed of gel matrix in the presence of the indicated concentrations of SHP2 or FGFR inhibitors. The growth of spheroids was further induced by addition of exogenous FGF2 (20 ng/ml). The area of the sphere 9 days after placement on the ECM was measured, and these values were normalized to the initial sphere size (Day 0). Data are the mean ± s.e.m. (*n* = 3) were ***p < 0.001.

We have previously reported that acquisition of resistance to lapatinib in HER2-transformed mammary epithelial cells (HME2) results in a mesenchymal phenotype that includes upregulation of FGFR1 and several ECM proteins, including fibronectin [10, 38]. Addition of SHP2 inhibitors to a low dose of the pan-ErbB inhibitor, neratinib, could significantly reduce the growth of the parental HER2-transformed cells (Supplementary Fig. S4). However, given our results in Fig. 3, we hypothesized that SHP2 also facilitates resistance to ErbB-targeted kinase inhibitors by facilitating bypass ECM and alternate RTK signaling. Therefore we first validated that our lapatinib resistant (LAPR) cells were independent of all ErbB signaling by demonstrating they were similarly resistant to neratinib (Fig. 4a). The LAPR cells also displayed elevated phosphorylation of SHP2 at Y542 compared to the drug sensitive parental cell line (Fig. 4b). Furthermore, pharmacological inhibition of SHP2 preferentially inhibited the growth of the LAPR cells as compared to the parental cells (Fig. 4c, d). Next, we focused on signaling recovery events following ErbB blockade. The addition of neratinib clearly prevented phosphorylation of HER2 in both the parental and LAPR cells, but downstream phosphorylation of AKT and ERK1/2 recovered quicker in LAPR cells compared to parental cells (Fig. 4e). These results are consistent with acquisition of bypass drivers of cell growth. To elucidate the mediators of ErbB bypass signaling we exposed LAPR cells to neratinib for 1 h, the drug was washed-off, and cells were subsequently allowed to recover in the presence or absence of compounds targeting SHP2 or FGFR for an additional 12 h (Fig. 4f). Following this transient treatment with neratinib recovery of AKT and ERK1/2 phosphorylation were delayed in the presence of SHP2 or FGFR inhibitors (Fig. 4f).

To further define the role of FGFR:SHP2 signaling in promoting the growth of drug resistant cells, we used a 3D tumor sphere assay. We recently described this assay, which consists of forming a large multicellular sphere of tumor cells using a non-adherent round bottom plate, followed by transferring the sphere to a bed of ECM to allow for tumor growth and invasion [39]. In these ECM-rich culture conditions, the growth of LAPR spheres was significantly increased by addition of exogenous FGF2. This FGF2-driven growth was abolished by inhibition of SHP2 or, as a control, direct targeting of FGFR kinase activity (Fig. 4g, h). These results indicate that in the presence of the ECM, SHP2 activity is required for FGFR signaling to facilitate HER2-independent cell growth and resistance to ErbB-targeted compounds.

### SHP2 facilitates signaling from multiple receptor tyrosine kinases

**Fig. 5:**
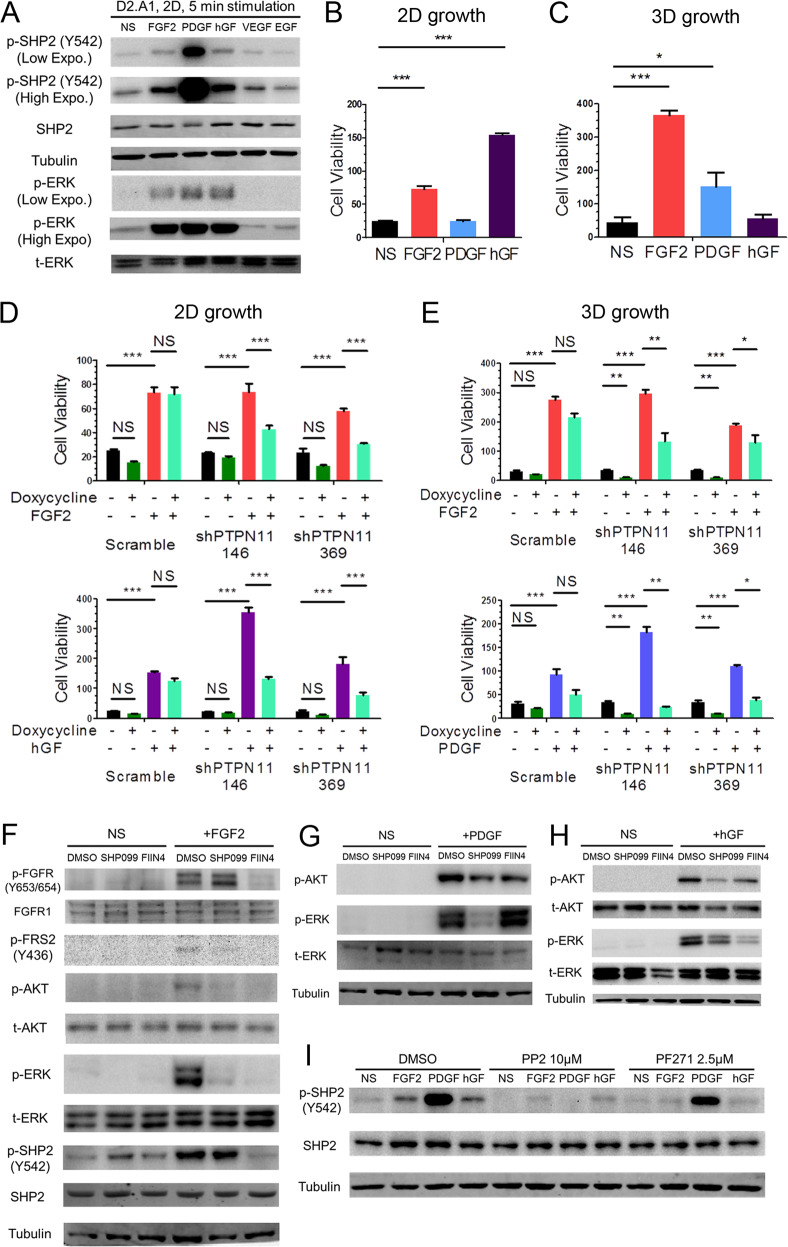
SHP2 facilitates signaling in response to several growth factors. **a** Immunoblot analyses showing the phosphorylation of SHP2 and ERK1/2 (ERK) induced by the indicated growth factors in D2.A1 cells. D2.A1 cell growth was induced by addition of exogenous FGF2, PDGF, and hGF in 2D (**b**) or 3D (**c**) culture for 6 days. D2.A1 cells expressing doxycycline inducible shRNAs targeting PTPN11 were treated with doxycycline in 2D culture (**d**) or 3D culture (**e**) in the presence of FGF2, hGF, or PDGF as indicated. For **b**–**e** cell viability was quantified as a relative bioluminescence ratio normalized to day 0. Data are the mean ± s.e.m. for at least three independent assays resulting in *p < 0.05, **p < 0.01, ***p < 0.001, or no significance (NS) as determined by a two-tail Student’s *t* test. **f–h** D2.A1 cells were pre-treated with SHP099 or FIIN4 for 24 h in serum-free media and cells were subsequently induced for 5 min with FGF2, PDGF, or hGF as indicated. Immunoblot analyses were used to detect phosphorylation of FGFR, FRS2, ERK1/2, AKT, and SHP2. **i** D2.A1 cells were pre-treated with PP2 or PF271 for 24 h in serum-free media, and cells were then induced for 5 min with FGF2, PDGF, or hGF. Immunoblot analyses were used to detect phosphorylation of SHP2. All immunoblots are representative of at least three independent experiments.

We next sought to investigate whether SHP2 facilitates proliferative signaling from additional RTKs that are also known to be involved in drug resistance and metastasis. We first detected the expression levels of multiple RTKs in several MBC cell lines and determined the ability of their cognate ligands to induce SHP2 phosphorylation (Supplementary Fig. S5). In the metastatic D2.A1 cells, transient addition of FGF2, platelet-derived growth factor (PDGF) and hepatocyte growth factor (hGF) could induce SHP2 phosphorylation at Y542 and downstream ERK1/2 phosphorylation (Fig. 5a). FGF2 induced the growth of D2.A1 cells under both 2D and 3D conditions while hGF and PDGF were only growth promoting under 2D and 3D culture conditions, respectively (Fig. 5b, c). Depletion of SHP2 significantly blocked cell growth induced by these specific growth factors (Fig. 5d, e). These results could also be recapitulated using SHP2-targeted inhibitors (Supplementary Fig. S6). To illustrate the downstream signaling events facilitated by SHP2, we performed ligand-induced signaling assays in the presence of SHP099. Phosphorylation of AKT and ERK1/2 induced by FGF2, PDGF, and hGF could all be reduced by pretreatment with SHP099 (Fig. 5f–h). Finally, unlike what we observed upon ECM-mediated signaling, growth factor-mediated phosphorylation of SHP2 could be readily blocked by the addition of PP2 (Fig. 5i).

### Combined targeting of SHP2 and FGFR improves therapeutic inhibition of metastasis

**Fig. 6:**
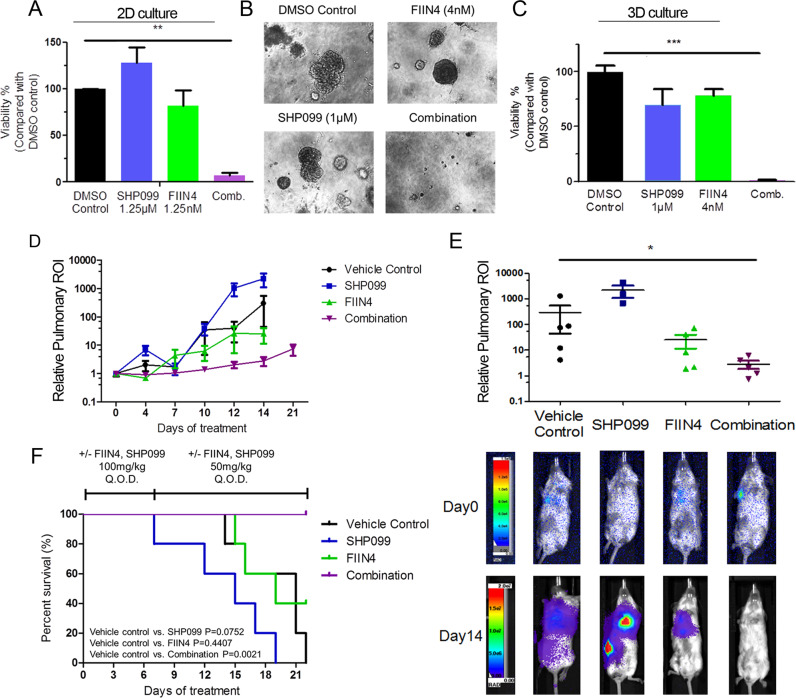
Inhibition of SHP2 synergizes with blockade of FGFR in vitro and in vivo. **a** 4T1 cells were seeded in 2D culture, and treated with the indicated concentrations of SHP099, FIIN4 alone, or both compounds for 6 days. **b, c** Single cell suspensions of 4T1 cells were plated in 3D matrices and treated with the indicated compounds alone or in combination for 12 days. Media containing DMSO was used as a vehicle control. Data in **a** and **c** data are bioluminescence values normalized to DMSO controls and are the mean ± s.e.m. of at least three independent experiments where **p < 0.01, ***p < 0.001 as determined by a two-tail Student’s *t* test. **d** Bioluminescence values for pulmonary regions of interest (ROI) from mice bearing 4T1 metastases normalized to values at the initiation of treatment. Data are the mean ± s.e.m. of 5 mice per treatment group. **e** (bottom) Representative bioluminescent images of metastasis and (top) quantified pulmonary ROI values from mice bearing 4T1 metastases 14 days after treatment initiation. Data are the mean ± s.e.m. of 5 mice per group resulting in *p < 0.05 as determined by a two-tail Mann Whitney test. **f** Differential survival analyses of tumor bearing mice in each treatment group, resulting in the indicated *p* values as determined by a log-rank test.

Our results thus far indicated that SHP2 is a shared node of both RTK and ECM signaling, suggesting there may be therapeutic potential for the use of SHP2 inhibitors in combination with targeted inhibition of a specific RTK. Therefore, we evaluated SHP099 in combination with the FGFR-targeted kinase inhibitor, FIIN4. Using concentrations of both compounds previously determined to be below effective dosages against the 4T1 cells resulted in complete blockade of cell growth when used in combination (Fig. 6a–c). Similar results were obtained with 11a-1 in 4T1 and other MBC cells (Supplementary Fig. S7). The synergy between SHP2 blockade and FGFR targeting was defined by establishing a combination index using COMPUSYN 1.0 for the 4T1 and other MBC cell lines (Supplementary Fig. S8). We also extended this combined therapeutic approach in vivo by treating late stage 4T1 metastases with SHP099:FIIN4 combination therapy. As opposed to our approach in Fig. 2, here mice were left untreated for 5 additional days following primary tumor removal to allow metastatic outgrowth to progress. Using this approach, we found that only the combination of SHP099 and FIIN4 was able to significantly delay late-stage metastatic progression and extend animal survival as compared to control (Fig. 6d–f). No significant weight loss was observed in the combination group, suggesting that this therapeutic protocol does not result in unacceptable toxicity (Supplementary Fig. S9). These data demonstrate the efficacy and feasibility of combining SHP2 inhibitory compounds with FGFR-targeted kinase inhibitors for the treatment of advanced breast cancer.

## Discussion

Drug-resistant progression of MBC can occur due to signaling events generated from multiple RTKs and can be further influenced by interactions with the ECM. This diversity in upstream inputs, and the downstream signaling pathways for which they activate, emphasizes the importance of targeting shared signaling nodes in the design of antimetastatic therapeutics. Although recent findings have begun to illustrate the oncogenic roles of SHP2 in breast cancer, mechanistic understanding of SHP2 signaling and the therapeutic value of targeting SHP2 in drug resistant MBC was still limited [30–32]. Herein, we demonstrate that SHP2 is indeed a shared signaling node that facilitates signaling from multiple RTKs as well as the ECM. Our work is supported by several other studies that clearly demonstrate SHP2 participates in RTK-mediated signaling [40–44].

**Fig. 7:**
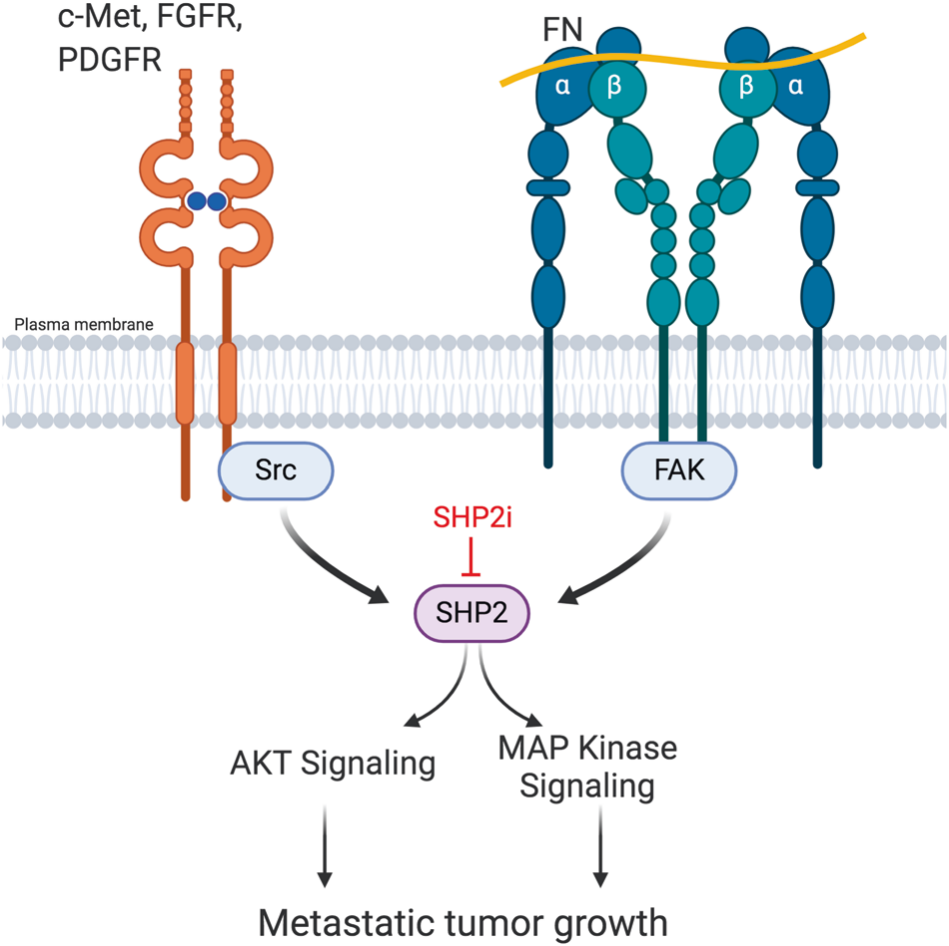
SHP2 is a shared node for ECM and RTK signaling. Activation of FAK via integrin engagement of ECM components drives SHP2 phosphorylation. Growth factor receptor signaling additionally contributes to phosphorylation of SHP2 via Src and FAK. SHP2 activity contributes to various downstream signaling pathways that facilitate metastatic tumor growth in the presence of currently used targeted therapies. Targeted inhibition of SHP2 (SHP2i), enhances responses to existing therapeutics. Figure was created using BioRender.

Using an inducible shRNA approach, we demonstrate that depletion of SHP2 specifically inhibited the ability of D2.A1 cells to grow under 3D culture conditions. These data are consistent with previous findings from our group and others that establish the requirement of β1 integrin:FAK signaling for D2.A1 cell growth in 3D culture [45, 46]. Consistent with these findings, culture of several additional MBC cell types under 3D conditions consistently induced SHP2 phosphorylation. Use of our recently-established tessellated scaffold culture system allowed for evaluation of specific ECM components, where we determined that fibronectin-mediated SHP2 phosphorylation occurs in a FAK-dependent manner. In contrast, SHP2 phosphorylation induced by various growth factors was reduced by the Src inhibitor PP2. These findings may serve to explain the clinical limitations observed in targeting either Src or FAK and suggest novel mechanisms by which ECM components interface with growth factor receptor signaling to support cell growth and survival (Fig. 7). Continued elucidation of upstream mechanisms of SHP2 phosphorylation remain important topics of research, but our findings herein clearly indicate that directed pharmacological targeting of SHP2 phosphatase activity is highly effective at inhibiting cell growth under 3D culture conditions. This is an unusual observation, as 3D culture typically diminishes cellular responses to targeted small molecule inhibitors. The ability of SHP2 inhibition to ablate matrix-mediated growth and survival signals indicate that SHP2 inhibitory compounds might synergize with RTK inhibitors or other therapeutics that particular tumors have an initial sensitivity to. We demonstrate this concept here using cell lines that we have previously demonstrated to initially respond to targeted inhibition of FGFR [10, 12, 35]. This concept is consistent with findings from other recent studies linking SHP2 function to acquisition of resistance of RAS mutant cancers to inhibition of MEK/ERK signaling [29, 33].

The importance of ECM-mediated phosphorylation of SHP2 is further emphasized by our findings indicating differential patient survival based on this biomarker. Clearly, SHP2 phosphorylation status, not mRNA expression levels, is the key determinant in patient prognosis. Current biomarkers that drive therapeutic indications are largely focused on determining genetic mutations or expression level changes of enzymes that are to be directly targeted with antibodies or small molecules. Targeting of signaling nodes, like SHP2, will require continued understanding of upstream inputs and downstream consequences and how they can be best utilized to identify the optimal patient population that will benefit from the addition of SHP2 therapy to other current standards of care.

Pharmacologic targeting of SHP2 remains an active area of research and discovery in the field of medicinal chemistry. Here we utilize a combination of genetic and pharmacological approaches to confidently elucidate the oncogenic functions of SHP2 in MBC. Both the SHP2 active site directed inhibitor 11a-1 and the allosteric inhibitor SHP099 phenocopied genetic depletion of SHP2, indicating that the observed pharmacologic effects are unlikely to be nonspecific. We did observe differences in outcomes between active site inhibition via 11a-1 versus allosteric inhibition via SHP099 in several of our assays. However, our studies fail to offer clear advantages of active site versus allosteric inhibition of SHP2. Several clinical trials are currently underway evaluating toxicity of allosteric inhibitors of SHP2 in various solid tumors (NCT03565003, NCT03634982). Further mechanistic studies comparing these modes of inhibition are required to better define their impact on SHP2 function and therapeutic outcome.

It is worthwhile to point out that our in vivo studies utilize two immune-competent mouse models. In addition to being expressed in epithelial cells, SHP2 phosphatase activity is also critical to T-cell function where it acts in a more traditional sense to facilitate immunoreceptor tyrosine-based inhibition motif signaling downstream of checkpoint receptors such as PD-1 and CTLA4 [47]. Given the importance of SHP2 in facilitating immune checkpoint signaling in cytolytic T-cells, our in vivo data using systemic administration of small molecule inhibitors could also reflect an enhancement of antitumor immunity [47, 48]. Indeed, recent studies suggest that systemic inhibition of FGFR increases recruitment of T-cells into tumors, enhancing response to antibody-mediated checkpoint blockade [49, 50]. Studies to further delineate the role of SHP2 inhibition in driving antimetastatic immunity through increased T-cell recruitment and checkpoint inhibition are currently underway in our laboratory.

In summary, we show that inputs from ECM and RTK signaling that contribute to drug resistance and metastasis are facilitated by SHP2 (Fig. 7). Our study pioneers the role of SHP2 in ECM signaling and presents pharmacological inhibition of SHP2 as a robust means to overcome matrix-mediated drug resistance. We also highlight the correlation of SHP2 Y542 phosphorylation status, not mRNA expression levels, to clinical prognosis in breast cancer patients. Finally, we elucidate a synergistic combination of SHP2 and FGFR inhibition in targeting MBC. Given the recent FDA approval of FGFR inhibitors and the current clinical evaluations of SHP2 targeted therapies, our studies strongly support development of this combined therapy for the treatment of MBC.

## Materials and methods

### Cell lines and culture conditions

The names and culture conditions of the cell lines in this study are listed in Supplementary Table S1. Luciferase expressing, 4T1, HMLE cells transformed by HER2 overexpression (HME2), their lapatinib resistant counterparts (LAPR), and the D2.A1 cells were constructed as we previously described [10, 12]. The other cell lines were purchased from ATCC. All cell lines were authenticated via the IDEXX IMPACT III CellCheck in December of 2018. All cell lines are regularly tested for mycoplasma contamination via PCR.

### Depletion of SHP2 with Dox-inducible shRNA

Dox-inducible eGFP-shRNA constructs with were purchased from Dharmacon. The targeting sequences are listed in Supplementary Table S2. The same vector with a scrambled non-targeting control shRNA was also purchased from Dharmacon. Lentiviral particles were produced in HEK-293 cells and were used to transduce 4T1 and D2.A1 cells, and stable integration was selected for using 5 μg/ml puromycin (Fisher). The stable cell lines were induced with doxycycline (Fisher) at 10 μg/ml for 3 days, and sorted for GFP positivity.

### Cell viability assays

The manufactures of the compounds used in in vitro assays are listed in Supplementary Table S3. 11a-1 was synthesized as previously described [27]. In 2D growth assays, the cells were seeded in 96-well plates with the indicated drug concentrations and cultured for 6 days. In 3D growth assays, 96-well plates were coated with 80% Cultrex (Sigma), which is a growth-factor-reduced 3D culture hydrogel matrix. Single cell suspensions were seeded on top of these hydrogel matrices in media containing 5% Cultrex and 10% FBS. In separate experiments 1 × 10^4^ tumor cells were formed into a macroscopic sphere via a 72 h plating in a non-adherent round bottom dish (Corning). These tumor spheres were similarly transferred to Cultrex matrices and tumor sphere growth and invasion were quantified by ImageJ 1.52a (NIH). Where indicated small molecules and growth factors were included upon seeding the tumor spheres onto the Cultrex and were refreshed every 3–4 days thereafter. The growth of luciferase-constructed cell lines was monitored by bioluminescent readings after addition of luciferin. Cell viability was also obtained using CellTiter-Glo Luminescent Cell Viability Assay (Promega) following the manufacturer’s instructions at the end points of assays.

### Immunoblotting

Immunoblotting of cells in 2D and 3D culture environments was executed as previously described [45, 51]. In the ligand-induced signaling assays, the cells were pretreated with inhibitors in serum-free media for 24 h prior to stimulation with FGF2 (20 ng/ml), PDGF (100 ng/ml), hGF (50 ng/ml), VEGF (100 ng/ml), and EGF (50 ng/ml). In the assays with tessellated scaffold culture system, the FN-coated scaffolds were prepared as previously described [38]. The cells were grown on scaffolds for 16 days, and treated with inhibitors for 24 h before harvesting. Primary and secondary antibodies used in this study are listed in Supplementary Table S4. Immunoblot results were collected and recorded using X-ray films, ChemiDoc Gel Imaging System (Bio-Rad) and LI-COR imaging (LI-COR Biosciences). Quantification of the blots was performed with ImageJ 1.52a (NIH).

### Animal care and in vivo metastatic models

For the 4T1 spontaneous metastasis model, 50k cells were engrafted onto the mammary fat pad via an intraductal injection. For the D2.A1 model, 1 × 10^6^ cells were injected via the lateral tail vein [12, 35]. All in vivo studies were conducted in 4–6 week old, female BALB/cJ mice purchased from Jackson Laboratories. All small molecule inhibitors were administered via oral gavage at the indicated concentrations and frequencies. Small molecule manufactures and gavage formulations are listed as Supplementary Table S3. Metastasis was monitored using bioluminescent imaging after intraperitoneal injection of luciferin using an AMI HT (Spectral Instruments). The lungs were fixed overnight by 10% formaldehyde (Fisher) immediately after sacrificing the mice and stored in 75% ethanol. Paraffin sectioning at 5 μm thickness and H&E staining were performed by AML Laboratories, Inc. (Jacksonville, FL). The photos of lung sections were acquired by Cytation 5 cell imaging multi-mode reader with Gen5 software (BioTek Instruments, Inc.). All in vivo assays were conducted under IACUC approval from Purdue University. No randomization or blinding was done.

### Drug combination study and data interpretation

The in vitro drug combination study was designed as described in the user guide of COMPUSYN 1.0 (ComboSyn Inc.; [52]). The small molecules FIIN4 and SHP099 were combined at a constant ratio.

### Clinical dataset analysis and code availability

For clinical outcomes, mRNA and Reverse Phase Protein Array (RPPA) raw data from Breast Invasive Carcinoma (BRCA) cohort of the Cancer Genome Atlas (TCGA) were downloaded from FireBrowse (www.firebrowse.org, Broad Institute of MIT and Harvard, MA). The RPPA data were first input into R, and the correlation plot of different genes was generated using the “*corrplot*” R package [53]. To analyze patient survival, we set 1500 days as the threshold of survival and then selected data from non-living patients. The patients were separated into two groups based on median expression levels. Finally, the survival curves were created by GraphPad Prism 5.0, and analyzed via a log-rank test. The detailed step-by-step methods and the R scripts for the correlation plot and data cleaning are included in the Supplementary information.

### Statistical analysis

A two tailed student’s *t* test was used for comparing differences between two groups of measurements in in vitro assays. Error bars show the standard error of the mean. IC_50_ values were calculated using GraphPad Prism 5.0 software via curve fit. Group measurements of the in vivo assays were compared with a Mann–Whitney non parametric test. Survival analysis was performed with GraphPad Prism 5.0 software, and the distributions of survival were compared by a log-rank test. No exclusion criteria were used in these studies, all statistical tests were appropriate as the groups being compared met the assumptions of the test and had similar variance.

## Supplementary information

Supplementary Figure 1

Supplementary Figure 2

Supplementary Figure 3

Supplementary Figure 4

Supplementary Figure 5

Supplementary Figure 6

Supplementary Figure 7

Supplementary Figure 8

Supplementary Figure 9

Supplementary Figure Legends

Supplementary Information

Supplementary Tables
