# Supplementary figures and images for: SHP2 is a multifunctional therapeutic target in drug resistant metastatic breast cancer
Source: Oncogene. 2020 Oct 8;39(49):7166–80. doi: 10.1038/s41388-020-01488-5 (PMC7714690; doi:10.1038/s41388-020-01488-5)

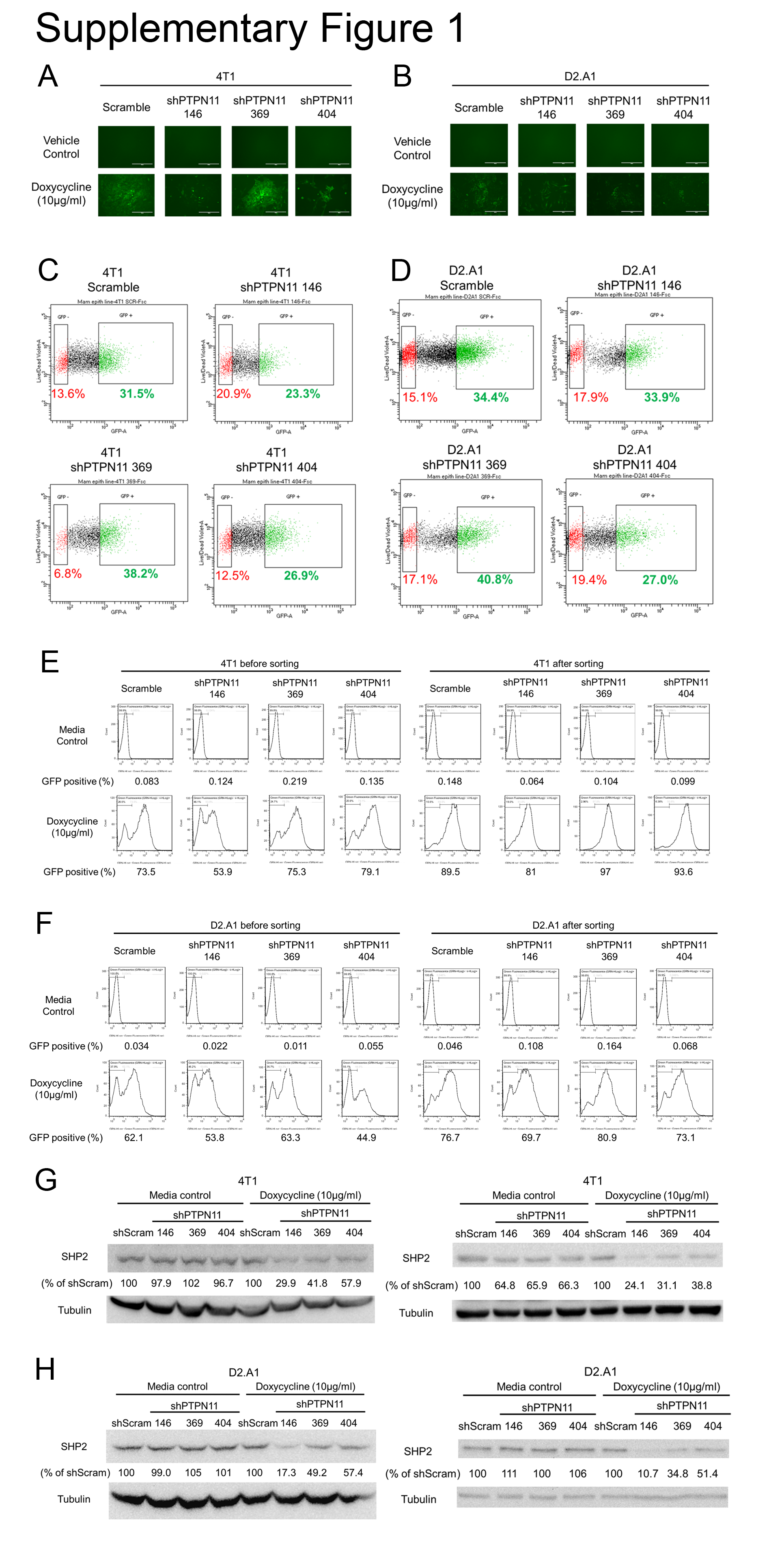

Supplement: Supplementary file 1 — Supplementary Figure 1 [file 41388_2020_1488_MOESM1_ESM.tif]

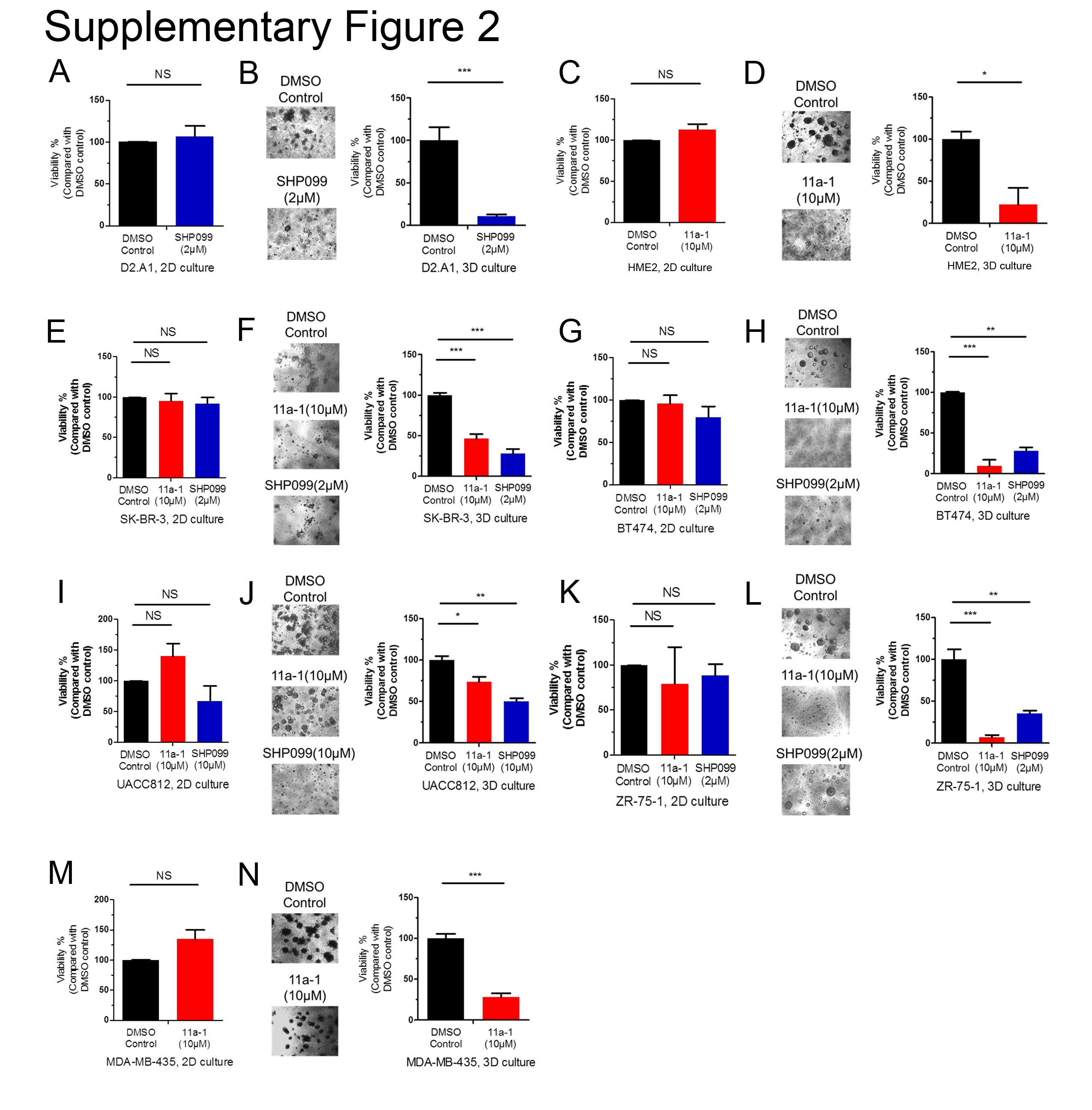

Supplement: Supplementary file 2 — Supplementary Figure 2 [file 41388_2020_1488_MOESM2_ESM.tif]

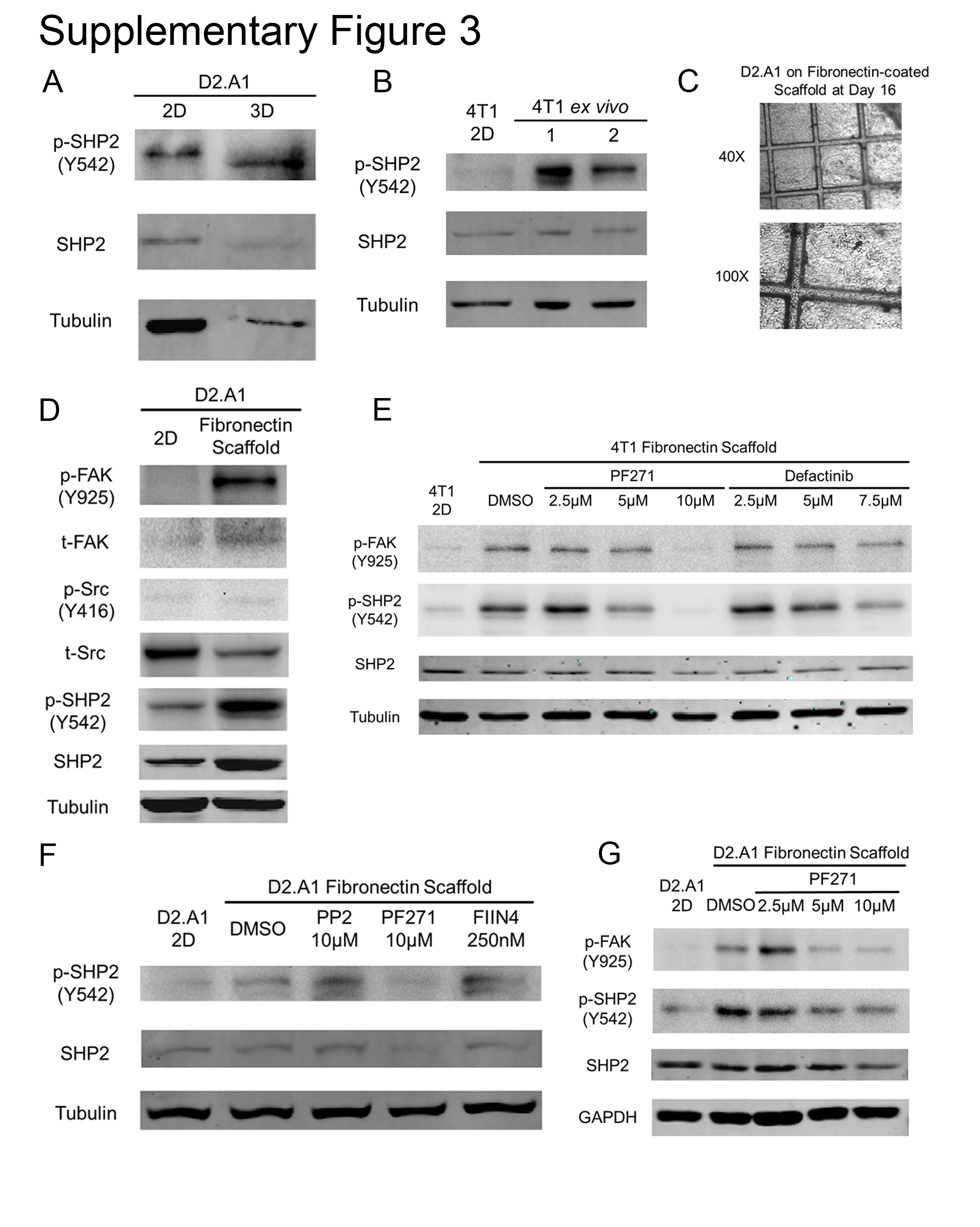

Supplement: Supplementary file 3 — Supplementary Figure 3 [file 41388_2020_1488_MOESM3_ESM.tif]

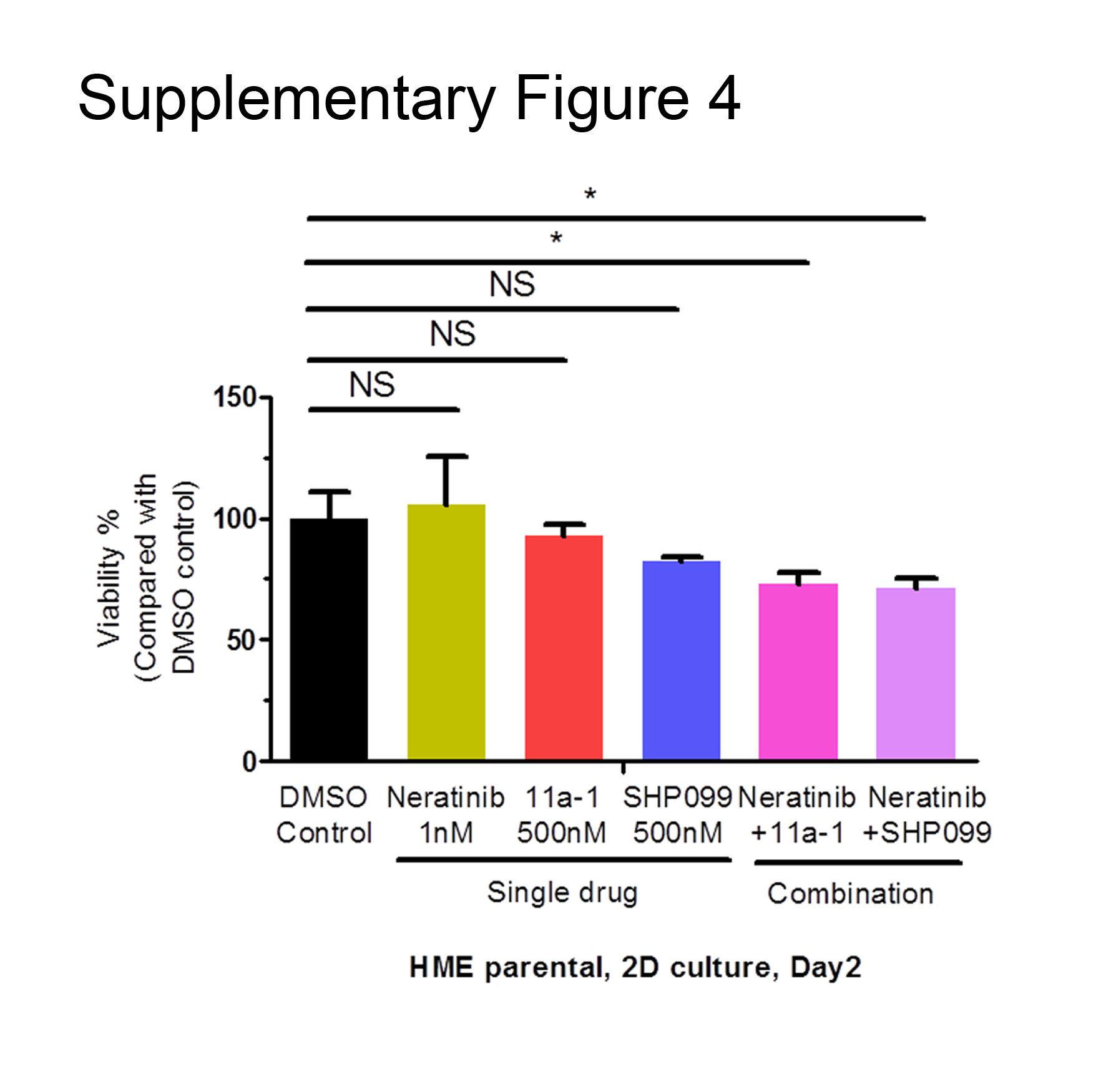

Supplement: Supplementary file 4 — Supplementary Figure 4 [file 41388_2020_1488_MOESM4_ESM.tif]

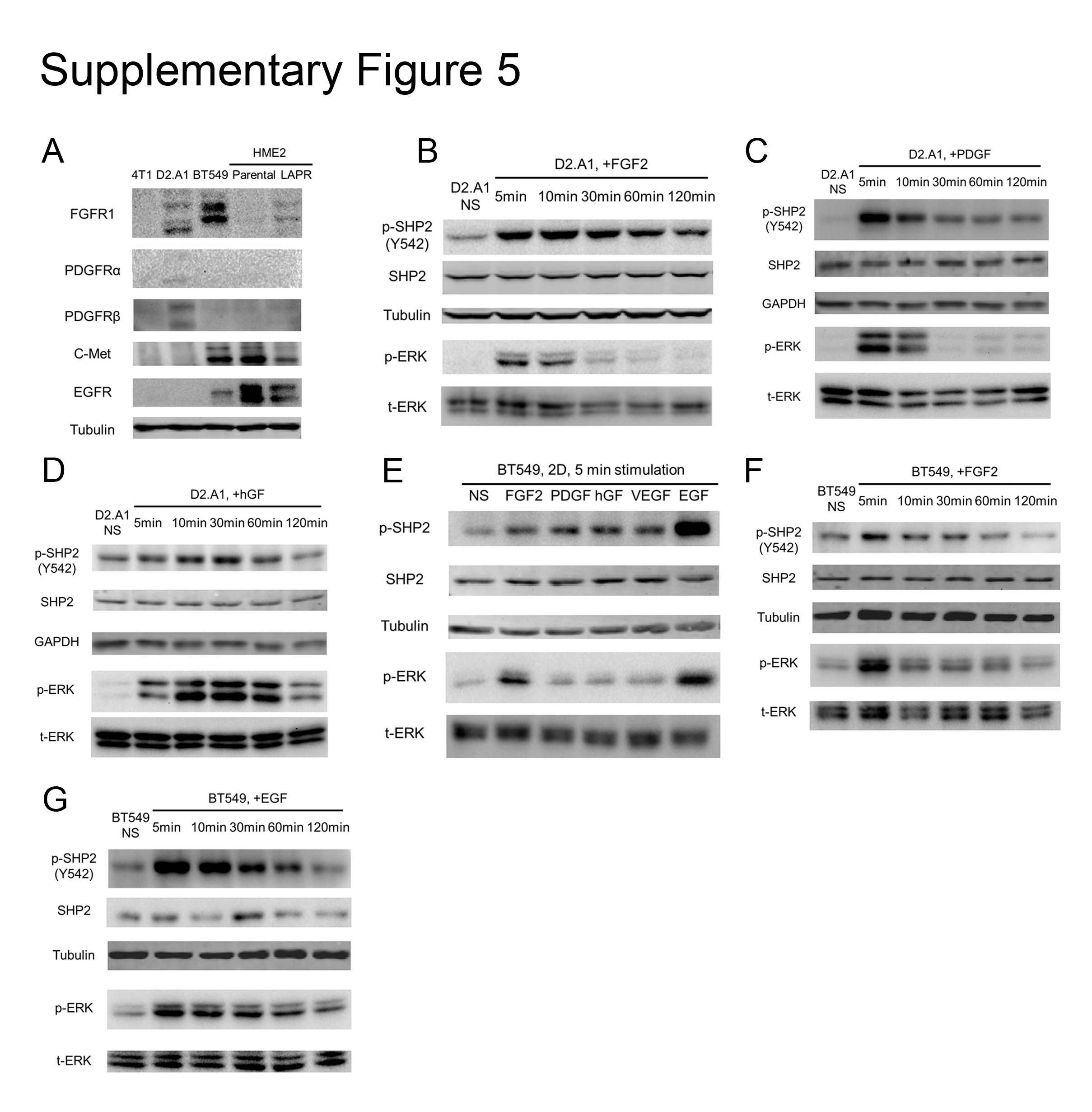

Supplement: Supplementary file 5 — Supplementary Figure 5 [file 41388_2020_1488_MOESM5_ESM.tif]

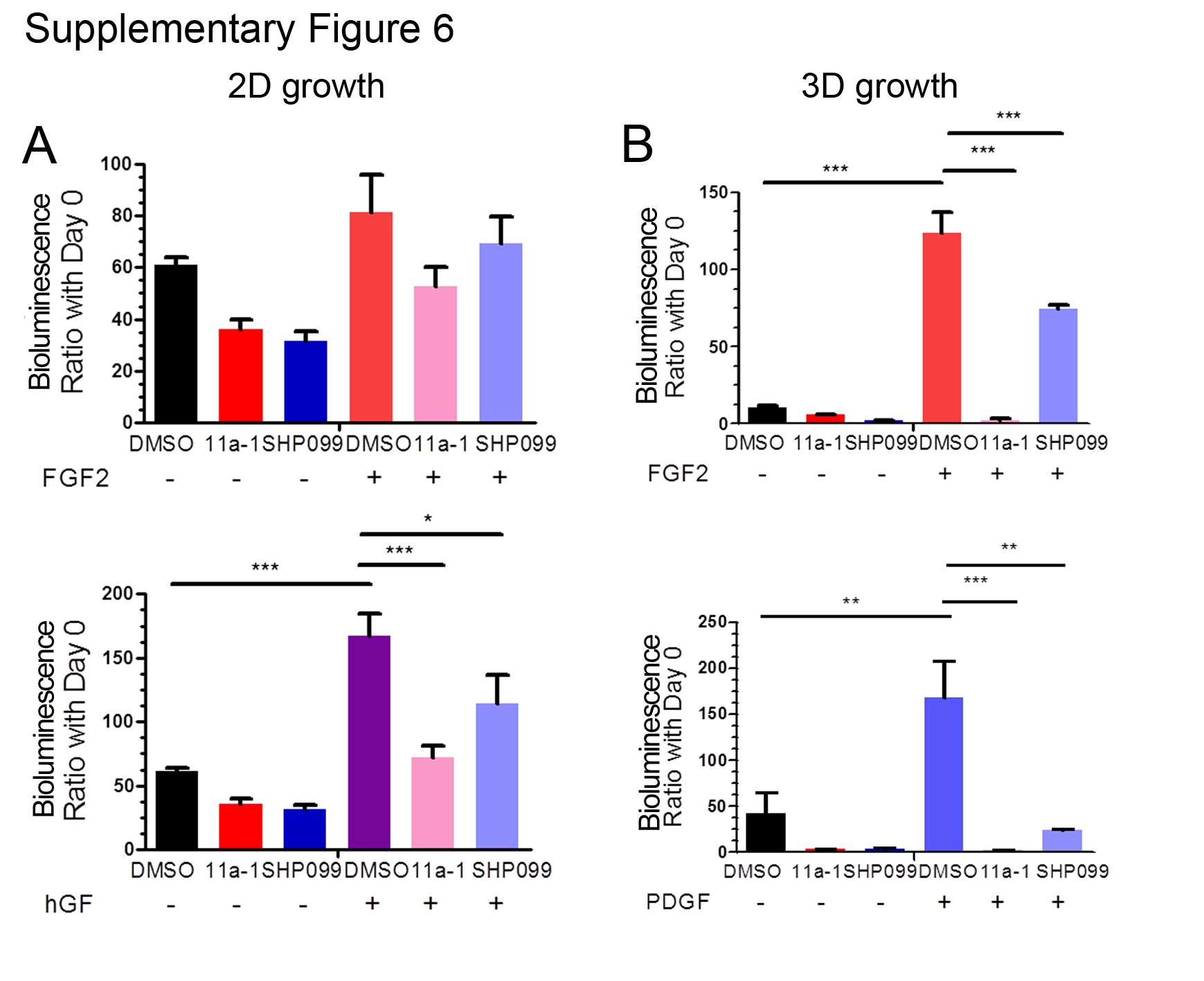

Supplement: Supplementary file 6 — Supplementary Figure 6 [file 41388_2020_1488_MOESM6_ESM.tif]

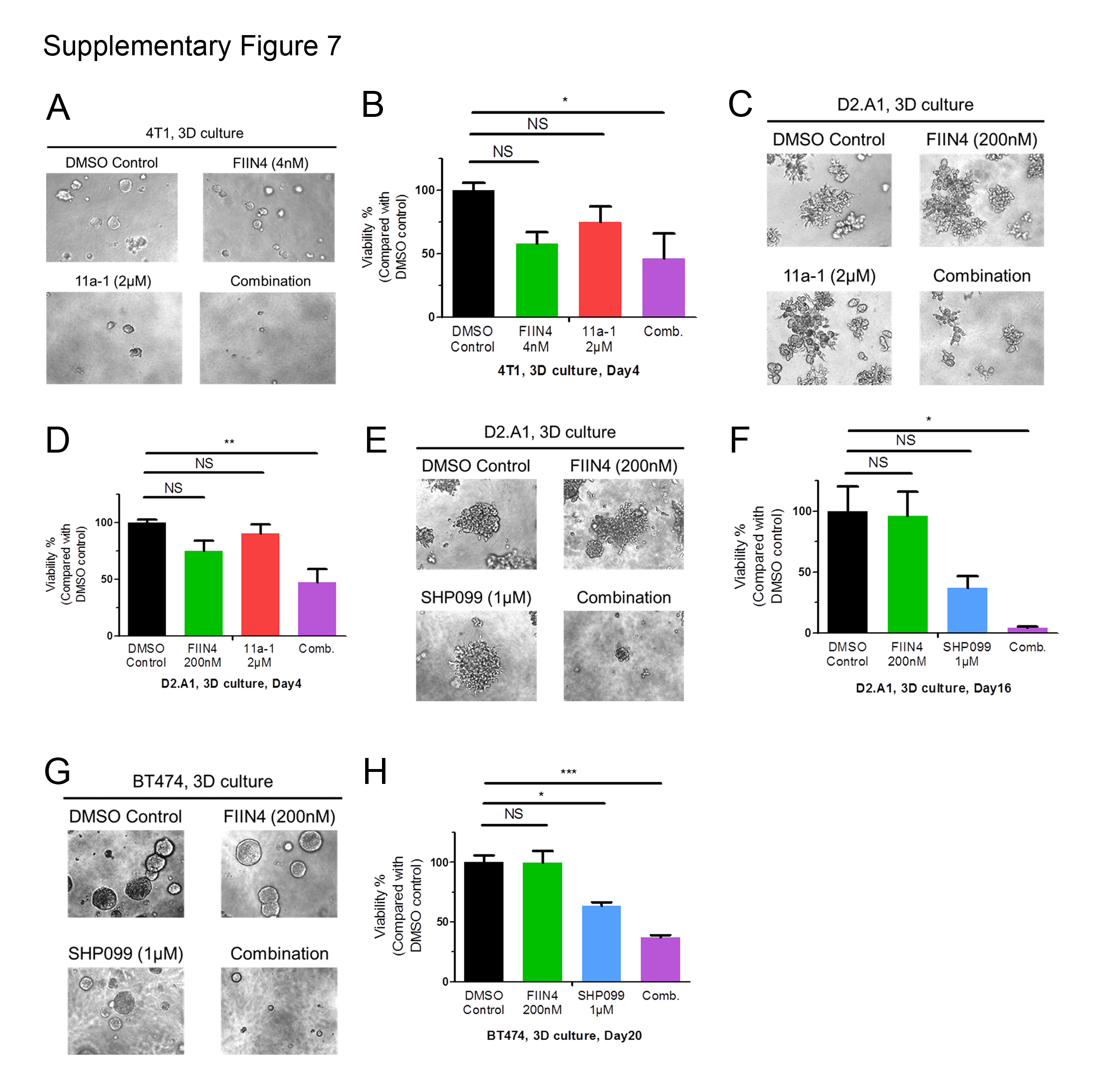

Supplement: Supplementary file 7 — Supplementary Figure 7 [file 41388_2020_1488_MOESM7_ESM.tif]

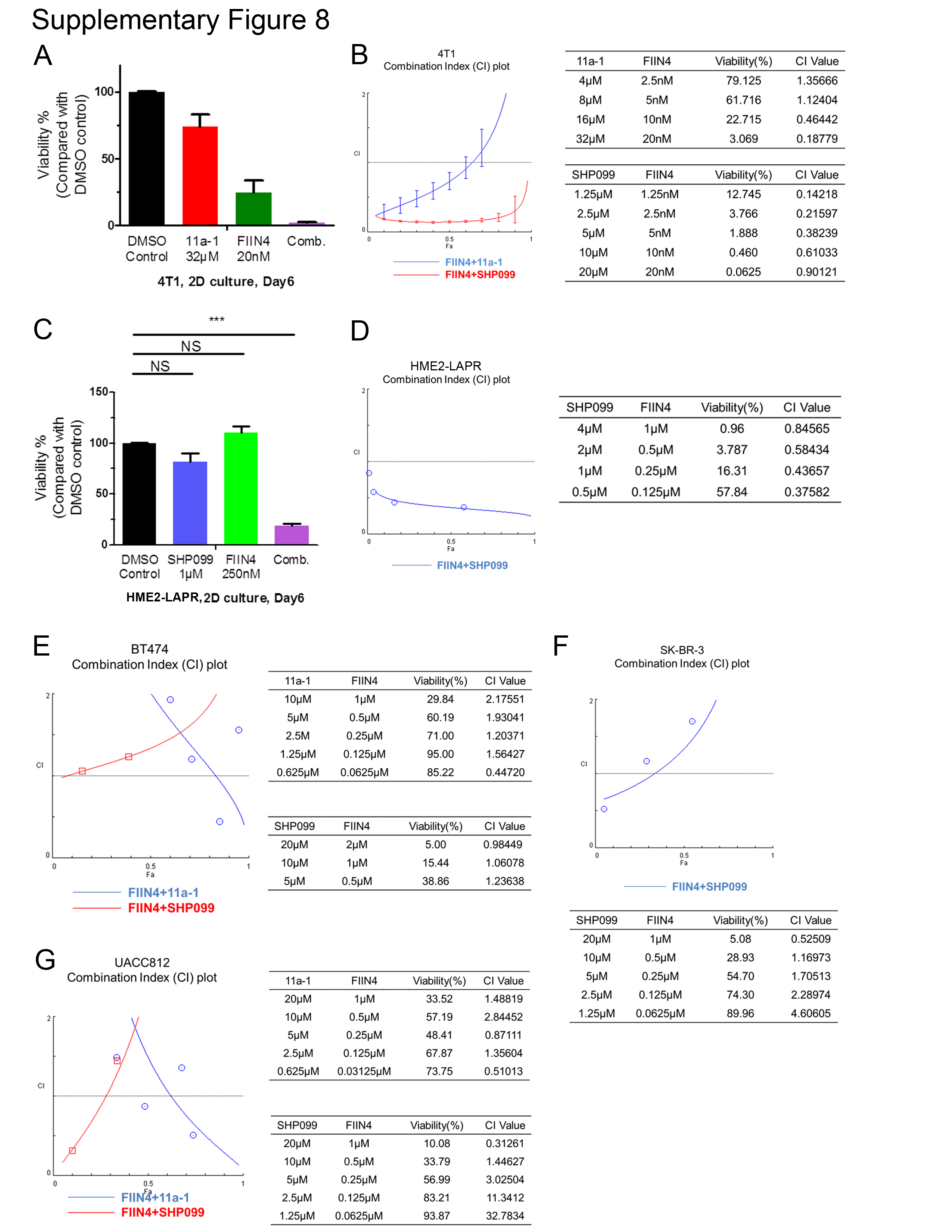

Supplement: Supplementary file 8 — Supplementary Figure 8 [file 41388_2020_1488_MOESM8_ESM.tif]

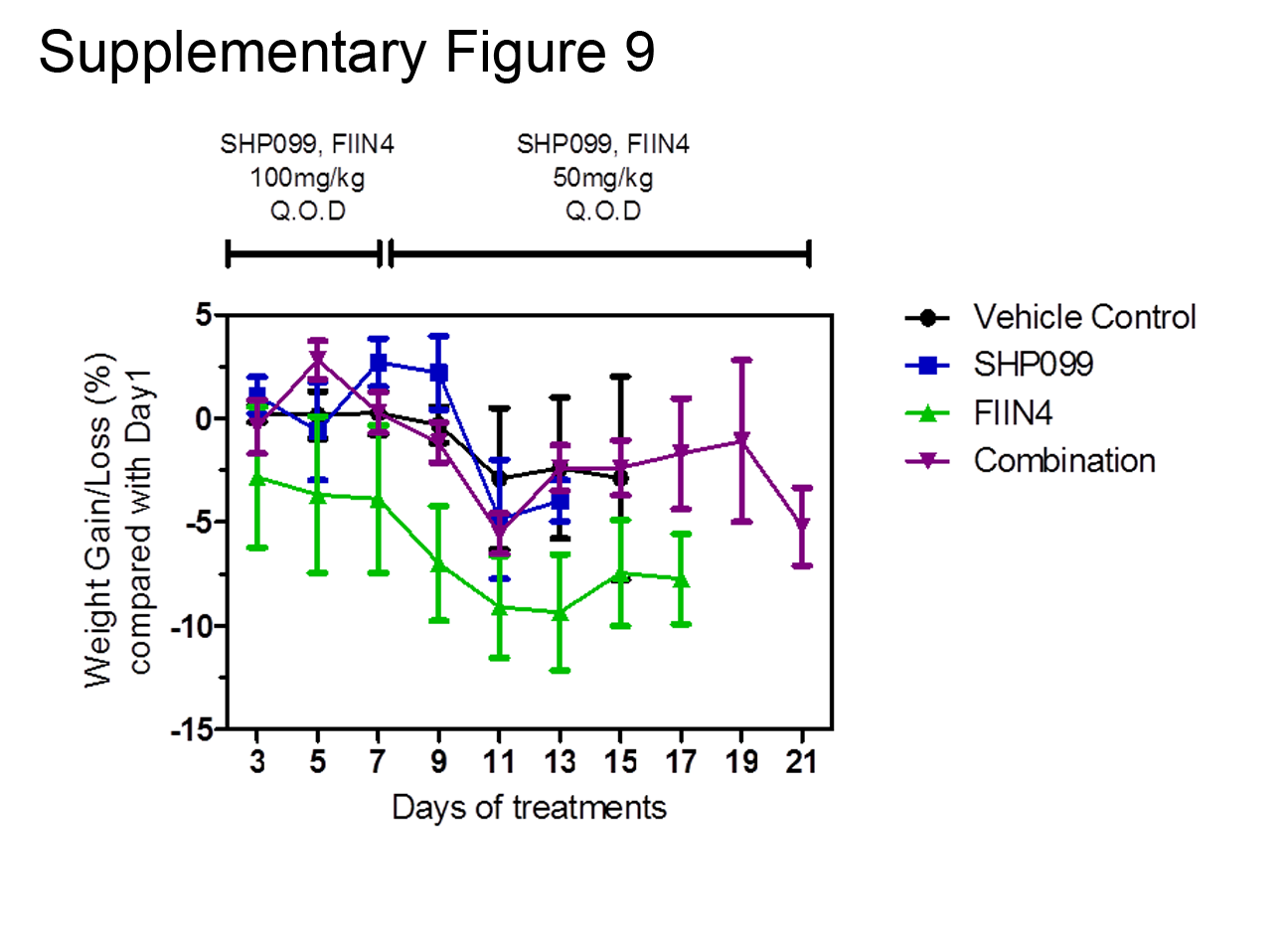

Supplement: Supplementary file 9 — Supplementary Figure 9 [file 41388_2020_1488_MOESM9_ESM.tif]
