# Supplementary material for: SHP2 is a multifunctional therapeutic target in drug resistant metastatic breast cancer
Source: Oncogene. 2020 Oct 8;39(49):7166–80. doi: 10.1038/s41388-020-01488-5 (PMC7714690; doi:10.1038/s41388-020-01488-5)
Supplement: Supplementary file 10 — Supplementary Figure Legends [file 41388_2020_1488_MOESM10_ESM.docx]

**Supplementary Figure Legends**

**Supplementary Fig. 1** Isolation of GFP^+^ cells upon doxycycline induction and validation of SHP2 depletion efficiencies. a, b Representative photomicrographs showing doxycycline induction of GFP^+^ 4T1 and D2.A1 cells stably expressing control and PTPN11-targeting shRNAs from the SMARTvector. c, d FACS sorting plots of 4T1 and D2.A1 cells transiently induced with doxycycline. GFP^-^ cells were highlighted as red dots, while GFP^+^ ones were highlighted with green. The corresponding percentage of GFP^-^ and GFP^+^ are listed on the plots. e, f Comparison of the GFP induction in the 4T1 and D2.A1 cultures before and after FACS for GFP^+^ cells. g, h Two independent repeats of immunoblot analyses with quantification for SHP2 in 4T1 and D2.A1 cells stably expressing three independent doxycycline-inducible shRNA sequences targeting PTPN11 with and without doxycycline induction, compared with scrambled (scram) shRNA controls.

**Supplementary Fig. 2** MBC cell lines are more sensitive to SHP2 inhibition under 3D culture conditions. Differential cell viability upon treatment with SHP099 or 11a-1 of the indicated cell lines cultured under 2D or 3D conditions. In all cases data are the mean ± s.e.m of cell viability normalized to DMSO control values, resulting in *p<0.05, **p<0.01, ***p<0.001 as determined via a two-tail Student’s t-test.

**Supplementary Fig. 3** Phosphorylation of SHP2 at Y542 is elevated under 3D culture conditions. a Immunoblot analyses showing the phosphorylation of SHP2 in D2.A1 cells cultured in 2D or 3D conditions. b Immunoblot analyses for phosphorylated SHP2 at Y542 in 2 separate 4T1 *ex vivo* cultures isolated from pulmonary metastases compared to *in vitro* cultured 4T1 cells in 2D conditions. c Representative photos showing D2.A1 cells cultured on FN-coated scaffolds. d Immunoblotting showing differential phosphorylation of FAK, Src and SHP2 in D2.A1 cells cultured on FN-coated scaffolds as compared to 2D culture. e Immunoblot analyses for phosphorylation of FAK at Y925 and SHP2 at Y542 in 4T1 cells cultured on FN-coated scaffolds treated with the indicated doses of PF271 or defactinib. f Immunoblot analyses showing the impact of PP2, PF271 and FIIN4 on the phosphorylation of SHP2 at Y542 in the D2.A1 cells cultured on FN-coated scaffolds. g Immunoblot analyses for phosphorylation of FAK at Y925 and SHP2 at Y542 in the D2.A1 cells cultured on FN-coated scaffolds treated with the indicated doses of PF271.

**Supplementary Fig. 4** Growth inhibitory effects of neratinib are enhanced when combined with SHP2 inhibitors in HME2 parental cells. HME2 parental cells were seeded in 96-well plates, and treated with the indicated concentrations of neratinib, SHP2 inhibitors, or both compounds for 2 days. Cell growth was quantified by relative luminescence ratio compared to untreated cells. (n = 4, resulting in *p<0.05, or no significance (NS) using a two-tail Student’s t-test).

**Supplementary Fig. 5** Dynamics of growth factor-induced SHP2 phosphorylation in MBC cell lines. a Immunoblot analyses showing the expression of FGFR1, PDGFRα, PDGFRβ, C-MET, and EGFR in the indicated cell lines. b-d Serum starved D2.A1 cells were induced by addition of exogenous FGF2 (B), PDGF (C) and hGF (D) in 2D culture for the indicated amounts of time. Cell lysates were analyzed by immunoblot for differential phosphorylation of SHP2 at Y542 and ERK1/2 (ERK). e Serum starved BT549 cells were induced by addition of the indicated growth factors and differential phosphorylation of SHP2 at Y542 and ERK1/2 (ERK) were analyzed by immunoblot. f-g Serum starved BT549 cells were induced by addition FGF2 (F) or EGF (G) in 2D culture for the indicated amounts of time. Cell lysates were analyzed by immunoblot for differential phosphorylation of SHP2 at Y542 and ERK1/2 (ERK).

**Supplementary Fig. 6** SHP2-targeted inhibition of growth factor-induced cell proliferation. a D2.A1 cells were stimulated with FGF2 (top graph) or hGF (bottom graph) in the presence or absence of SHP2 inhibitors in 2D culture. b D2.A1 cells were treated with SHP2 inhibitors in 3D culture in the presence or absence of FGF2 (top graph) or PDGF (bottom graph). In all cases data are the mean cell growth values quantified as relative bioluminescence normalized to day 0. (n = 3, resulting in **p<0.01, ***p<0.001, using a two-tail Student’s t-test).

**Supplementary Fig. 7** Combined blockade of SHP2 and FGFR enhances growth inhibition of MBC cells. 4T1 cells (a, b), D2.A1 cells (c-f) and BT474 cells (g, h) were plated under 3D culture conditions and treated with the indicated compounds alone or in combination. Media containing DMSO was used as a vehicle control. Representative photos are shown, and quantitative data are the mean ±s.e.m. cell viability measurements (n=3) normalized to vehicle controls resulting in; no significance (NS), *p<0.05, **p<0.01, ***p<0.001 as determined by a two-tail Student’s t-test,.

**Supplementary Fig. 8** Inhibition of SHP2 synergizes with FGFR inhibition in MBC cells. a 4T1 cells were seeded in 2D culture, and treated with 11a-1, FIIN4 or a combination of the two compounds for 6 days. Media containing DMSO was used as a vehicle control. Cell viability was monitored using cell titer glo assays and data are the mean ±SD values normalized to the vehicle control. b Combination Index (CI) plots and charts of 4T1 cells treated with SHP2 inhibitors and FIIN4 generated by COMPUSYN 1.0. c HME2-LAPR cells were seeded in 2D culture, and treated with SHP-099, FIIN4 or a combination of the two compounds for 6 days. Media containing DMSO was used as a vehicle control. Cell viability was monitored using cell titer glo assays and data are the mean (n=3) ±SD values normalized to the vehicle control resulting in No Significance (NS) or ***p<0.001 as determined by a two-tail Student’s t-test. d-g Combination Index (CI) plots and charts of SHP2 inhibitors and FIIN4 generated by COMPUSYN 1.0 in HME2-LAPR, BT474, SK-BR-3 and UACC812 cells, according to the corresponding 2D growth data in these cells.

**Supplementary Fig. 9** Combined administration of SHP099 and FIIN4 does not enhance weight loss. Mice bearing 4T1 metastases were treated with SHP099 and FIIN4 alone or in combination as indicated. Data are the mean, ±s.d., body weights (n=5) normalized to day 1 of treatment.
