# Supplementary material for: SHP2 is a multifunctional therapeutic target in drug resistant metastatic breast cancer
Source: Oncogene. 2020 Oct 8;39(49):7166–80. doi: 10.1038/s41388-020-01488-5 (PMC7714690; doi:10.1038/s41388-020-01488-5)
Supplement: Supplementary file 11 — Supplementary Information [file 41388_2020_1488_MOESM11_ESM.docx]

**Supplementary information**

*Step-by-step methods for correlation plot*

1. Open <http://firebrowse.org/> with browser, select ‘Breast invasive carcinoma (BRCA)’ cohort.
2. Click ‘Reverse Phase Protein Array’ tab, and click ‘RPPA_AnnotateWithGene (MD5)’ to download data package.
3. Open ‘BRCA.rppa.txt’ in the downloaded data package, and select all the information in the file.
4. Copy and paste the information in the ‘BRCA.rppa.txt’ to Microsoft EXCEL, and save as csv document type.
5. Search in Microsoft EXCEL for the interested gene names: ‘PTPN11|SHP-2_pY542’, ‘SRC|Src’, ‘SRC|Src_pY416’, ‘SRC|Src_pY527’, ‘STAT3|STAT3_pY705’, ‘EGFR|EGFR’, ‘EGFR|EGFR_pY1068’, ‘EGFR|EGFR_pY1173’, ‘ERBB2|HER2’ ‘ERBB2|HER2_pY1248’, ‘MET|c-Met’, ‘MET|c-Met_pY1235’,and copy these rows and paste as Transpose to a new Microsoft EXCEL file, and save as rppa.csv.
6. Run the R scripts for correlation plots in R studio, and choose rppa.csv as the input.
7. The correlation plot will be generated, and save the plot.

*R scripts for correlation plots*

rm(list = ls())

x<-read.csv(file.choose()) #Choose rppa.csv here.

library(corrplot)

M <- cor(x)

library(RColorBrewer)

corrplot(M, method = "circle", type="upper",

col = brewer.pal(n=10,name = "RdBu"))

*Step-by-step methods for survival plot with RPPA data*

1. Open <http://firebrowse.org/> with browser, select ‘Breast invasive carcinoma (BRCA)’ cohort.
2. Click ‘Reverse Phase Protein Array’ tab, and click ‘RPPA_AnnotateWithGene (MD5)’ to download data package.
3. Open ‘BRCA.rppa.txt’ in the downloaded data package, and select all the information in the file.
4. Copy and paste the information in the ‘BRCA.rppa.txt’ to Microsoft EXCEL, and save as raw_rppa.csv.
5. Click ‘Clinical’ tab, and click ‘Merge_Clinical (MD5)’ to download data package.
6. Open ‘BRCA.clin.merged.txt’ in the downloaded data package, and select all the information in the file.
7. Copy and paste the information in the ‘BRCA.clin.merged.txt’ to Microsoft EXCEL.
8. Search in the Microsoft EXCEL for the key word ‘patient.samples.sample.portions.shipment_portion.shipment_portion_bcr_aliquot_barcode’ , rename the cell as ‘Composite.Element.REF’.
9. Cut this row and paste as the first row of the table, and save the file as clinic.csv.
10. Run the R scripts for merging clinic data with RPPA data in R studio, and choose raw_rppa.csv and clinic.csv as the input.
11. Open the output c.csv in Microsoft EXCEL, and search for the rows named‘PTPN11|SHP-2_pY542’ and ‘patient.days_to_death’.
12. Copy these rows and paste as Transpose to a new Microsoft EXCEL file, sort the data according the ‘PTPN11|SHP-2_pY542’, delete all the rows with non-numeric values.
13. Determine the median of ‘PTPN11|SHP-2_pY542’, and separate the data as two groups.
14. Plot under the threshold of survival with GraphPad Prism 5.0.

*R scripts for merging clinic data with RPPA data*

rm(list = ls())

clinic<-read.csv(choose.files()) #Choose clinic.csv here.

RPPA<-read.csv(choose.files()) #Choose raw_rppa.csv here.

library("dplyr")

a<-merge(clinic,RPPA,all=TRUE)

b<-merge(RPPA,clinic,all=TRUE)

c<-union(a,b)

write.csv(c,"D:/c.csv")

*Step-by-step methods for survival plot with mRNA data*

1. Open <http://firebrowse.org/> with browser, select ‘Breast invasive carcinoma (BRCA)’ cohort.
2. Click ‘mRNA’ tab, and click ‘mRNA_Preprocess_Median (MD5)’ to download data package.
3. Open ‘BRCA.medianexp.txt’ in the downloaded data package, and select all the information in the file.
4. Copy and paste the information in the ‘BRCA.medianexp.txt’ to Microsoft EXCEL, and save as raw_mrna.csv.
5. Click ‘Clinical’ tab, and click ‘Merge_Clinical (MD5)’ to download data package.
6. Open ‘BRCA.clin.merged.txt’ in the downloaded data package, and select all the information in the file.
7. Copy and paste the information in the ‘BRCA.clin.merged.txt’ to Microsoft EXCEL.
8. Search in the Microsoft EXCEL for the key word ‘patient.samples.sample.portions.portion.analytes.analyte-2.aliquots.aliquot-2.bcr_aliquot_barcode’ , rename the cell as ‘Hybridization REF’.
9. Cut this row and paste as the first row of the table, and save the file as clinic2.csv.
10. Run the R scripts for merging clinic data with RPPA data in R studio, and choose raw_mrna.csv and clinic2.csv as the input.
11. Open the output d.csv in Microsoft EXCEL, and search for the rows named‘PTPN11’ and ‘patient.days_to_death’.
12. Copy these rows and paste as Transpose to a new Microsoft EXCEL file, sort the data according the ‘PTPN11’, delete all the rows with non-numeric values.
13. Determine the median of ‘PTPN11’, and separate the data as two groups.
14. Plot under the threshold of survival with GraphPad Prism 5.0.

*R scripts for merging clinic data with mRNA data*

rm(list=ls())

Clinic<-read.csv(choose.files()) #Choose clinic2.csv here.

mRNA<-read.csv(choose.files()) #Choose raw_mrna.csv here.

library("dplyr")

a<-merge(Clinic,mRNA,all=TRUE)

b<-merge(mRNA,Clinic,all=TRUE)

d<-union(a,b)

write.csv(c,"D:/d.csv")
