# Supplementary material for: SHP2 is a multifunctional therapeutic target in drug resistant metastatic breast cancer
Source: Oncogene. 2020 Oct 8;39(49):7166–80. doi: 10.1038/s41388-020-01488-5 (PMC7714690; doi:10.1038/s41388-020-01488-5)
Supplement: Supplementary file 12 — Supplementary Tables [file 41388_2020_1488_MOESM12_ESM.docx]

**Supplementary Table 1 Cell lines and culture conditions in this study**

| Name of cell line | Culture condition |
| --- | --- |
| 4T1 (ATCC^®^ CRL-2539™) | DMEM with 10% Fetal Bovine Serum (FBS) |
| D2.A1 | DMEM with 10% Fetal Bovine Serum (FBS) |
| HME2 parental | DMEM with 10% Fetal Bovine Serum (FBS) and 0.1% Human Insulin solution |
| HME2 LAPR | DMEM with 10% Fetal Bovine Serum (FBS) and 0.1% Human Insulin solution |
| BT-474 (ATCC^®^ HTB-20™) | DMEM with 10% Fetal Bovine Serum (FBS) |
| HEK-293 (ATCC^®^ CRL-1573™) | DMEM with 10% Fetal Bovine Serum (FBS) |
| MDA-MB-435S (ATCC® HTB-129™) | DMEM with 10% Fetal Bovine Serum (FBS) |
| SK-BR-3 (ATCC^®^ HTB-30™) | DMEM with 10% Fetal Bovine Serum (FBS) |
| UACC-812 (ATCC^®^ CRL-1897™) | DMEM with 10% Fetal Bovine Serum (FBS) and 32ng/ml EGF |
| ZR-75-1 (ATCC^®^ CRL-1500™) | RPMI-1640 with 10% Fetal Bovine Serum (FBS) |
| BT-549 (ATCC^®^ HTB-122™) | RPMI-1640 with 10% Fetal Bovine Serum (FBS) and 0.1% Human Insulin solution |
|  |  |

**Supplementary Table 2 Targeting sequences of doxycycline inducible shRNA vectors**

| Simplified ID | Source Clone ID | Target | Vector | Targeting Sequence |
| --- | --- | --- | --- | --- |
| 146 | V3IMMMCG_14469146 | Ptpn11 3’-UTR | piSMART mCMV/TurboGFP | AGACTAGACGAGCGTTCCC |
| 369 | V3IMMMCG_11150369 | Ptpn11 CDS | piSMART mCMV/TurboGFP | ACAGAAGCACAGTACCGGT |
| 404 | V3IMMMCG_15981404 | Ptpn11 CDS | piSMART mCMV/TurboGFP | ATCGCGGAGATGGTTTCAC |

**Supplementary Table 3 Drugs and reagents**

| Drug / Reagent | Source | Identifier / formulation |
| --- | --- | --- |
| SHP099 dihydrochloride (*in vitro* study) | Selleck | Catalog No: S8278 |
| SHP099 dihydrochloride (*in vivo* grade) | Chemietek | Catalog No: CT-SHP099  Formulation: 0.5% Hydroxypropyl Methylcellulose |
| FIIN4 | Achemtek | Catalog No: 0107-000063  Formulation: 0.5% Carboxymethylcellulose |
| Neratinib (HKI-272) | Selleck | Catalog No: S2150 |
| PP2 | Selleck | Catalog No: S7008 |
| PF-562,271 (PF271) | Pfizer | Under agreement from Pfizer Inc. |
| defactinib (VS-6063) | Selleck | Catalog No: S7654 |
| Basic FGF (FGF2), Human | GoldBio | Catalog No: 1140-02-10 |
| Recombinant Human PDGF-BB Protein, CF | R&D systems | Catalog No: 220-BB-010 |
| Recombinant Mouse HGF Protein, CF | R&D systems | Catalog No: 2207-HG/CF |
| Recombinant Mouse VEGF 164 Protein, CF | R&D systems | Catalog No: 493-MV-005/CF |
| EGF, Human | GoldBio | Catalog No: 1150-04-100 |
| Cultrex^®^ RGF BME PathClear^®^ | Sigma | Catalog No: 3433-005-01  Concentration: 17.05 mg/ml |
| D-Luciferin, Potassium Salt | GoldBio | Catalog No: LUCK-100 |

**Supplementary Table 4 Primary and secondary antibodies used in this study**

| Antibody | Source | Identifier | Host |
| --- | --- | --- | --- |
| Anti-SHP2 (phospho Y542) antibody [EP508(2)Y] | Abcam | Catalog No: ab62322 | Rabbit |
| SH-PTP2 Antibody (B-1) | Santa Cruz Biotechnology | Catalog No: sc-7384 | Mouse |
| Phospho-FRS2-α (Tyr436) Antibody | Cell Signaling Technology | Catalog No: #3861 | Rabbit |
| Phospho-FAK (Tyr925) Antibody | Cell Signaling Technology | Catalog No: #3284 | Rabbit |
| FAK Recombinant Rabbit Monoclonal Antibody (5H18L19) | Invitrogen | Catalog No: 701094 | Rabbit |
| Phospho-Src Family (Tyr416) Antibody | Cell Signaling Technology | Catalog No: #2101 | Rabbit |
| Phospho-Src (Tyr527) Antibody | Cell Signaling Technology | Catalog No: #2105 | Rabbit |
| Src Antibody | Cell Signaling Technology | Catalog No: #2108 | Rabbit |
| Phospho-Akt (Ser473) Antibody | Cell Signaling Technology | Catalog No: #9271 | Rabbit |
| Akt Antibody | Cell Signaling Technology | Catalog No: #9272 | Rabbit |
| Phospho-p44/42 MAPK (Erk1/2) (Thr202/Tyr204) Antibody | Cell Signaling Technology | Catalog No: #9101 | Rabbit |
| p44/42 MAPK (Erk1/2) Antibody | Cell Signaling Technology | Catalog No: #9102 | Rabbit |
| FGF Receptor 1 (D8E4) XP^®^ Rabbit mAb | Cell Signaling Technology | Catalog No: #9740 | Rabbit |
| Phospho-HER2/ErbB2 (Tyr1196) (D66B7) Rabbit mAb | Cell Signaling Technology | Catalog No: #6942 | Rabbit |
| HER2/ErbB2 Antibody | Cell Signaling Technology | Catalog No: #2242 | Rabbit |
| Phospho-FGF Receptor (Tyr653/654) Antibody | Cell Signaling Technology | Catalog No: #3471 | Rabbit |
| PDGF Receptor α (D1E1E) XP^®^ Rabbit mAb | Cell Signaling Technology | Catalog No: #3174 | Rabbit |
| PDGF Receptor β (28E1) Rabbit mAb | Cell Signaling Technology | Catalog No: #3169 | Rabbit |
| Met Antibody | Cell Signaling Technology | Catalog No: #4560 | Rabbit |
| Tubulin, beta | Developmental Studies Hybridoma Bank (DSHB) | Catalog No: E7 | Mouse |
| GAPDH (2D4A7) | Santa Cruz Biotechnology | Catalog No: sc-59541 | Mouse |
| Goat anti-Mouse IgG (H+L) Secondary Antibody, HRP | ThermoFisher | Catalog No: 62-6520 | Goat |
| Goat anti-Rabbit IgG (H+L) Secondary Antibody, HRP | ThermoFisher | Catalog No: 65-6120 | Goat |
| IRDye^®^ 680RD Goat anti-Mouse IgG Secondary Antibody | LI-COR, Inc. | P/N No: 926-68070 | Goat |
| IRDye® 800CW Goat anti-Rabbit IgG Secondary Antibody | LI-COR, Inc. | P/N No: 926-32211 | Goat |
